# Improvement of the production of biomass and lipid by *Papiliotrema laurentii* combining flux balance analysis and central composite rotational design

**DOI:** 10.1007/s00449-026-03311-z

**Published:** 2026-03-23

**Authors:** Samuel Lessa Barbosa, Eduardo Luís Menezes de Almeida, Rafaela Zandonade Ventorim, Jimmy Soares, Wendel Batista da Silveira

**Affiliations:** Laboratory of Microbial Physiology, Department of Microbiology, Institute of Biotechnology Applied To Agriculture, Universidade Federal de Viçosa, Viçosa, Minas Gerais Brazil

**Keywords:** Circular economy, Central Compound Rotational Design, Volumetric oxygen mass transfer coefficient (*k*_*L*_*a*), Biomass optimization, Bioreactor cultivation, Sustainable oleochemicals

## Abstract

The growing demand for oleochemicals, currently derived from edible oils, raises sustainability concerns due to competition for agricultural resources. Oleaginous yeasts are an alternative oil source. Among them, *Papiliotrema laurentii* stands out due to rapid lipid accumulation and high lipid contents. Nonetheless, the biomass and lipid titers by this yeast are low in the culture media used for oleaginous yeasts. Herein, we combined Flux Balance Analysis (FBA) and Central Composite Rotational Design (CCRD) to identify carbon and nitrogen sources that favour both biomass and lipid production by *P. laurentii*. Based on biomass yield predicted by FBA, we identified lactose and urea as suitable carbon and nitrogen sources, respectively. We also used a CCRD to evaluate the effects of carbon-to-nitrogen (C:N) ratio, volumetric oxygen mass transfer coefficient (*k*_*L*_*a*), and inoculum on biomass and lipid production. Optimal conditions for biomass production were: initial OD_600_ of 0.8, a C:N ratio of 63:1, and a *k*_*L*_*a* of 28.41 h^−1^, yielding 10.82 g/L of biomass. For lipid titer, optimal conditions were: initial OD_600_ of 0.1, a C:N ratio of 100:1, and a *k*_*L*_*a* of 28.41 h^−1^, resulting in 3.48 g/L of lipids. Bioreactor cultivations showed that increasing *k*_*L*_*a* to 49.42 h^−1^ significantly enhanced both lipid content and titer. Under optimized conditions, increases of 691% in biomass and 815% lipid titer were recorded. Thus, this is the first study systematically optimizing cultivation conditions of *P. laurentii* combining FBA and CCRD.

**Supplementary Information:**

The online version contains supplementary material available at 10.1007/s00449-026-03311-z.

## Introduction

The demand for oleochemicals such as fatty acids, fatty alcohols and fatty methyl esters, which are mainly produced from edible oils, has increased over the last years [1]. The can be applied for example for the production of cosmetics, biofuels and lubricants [2–4]. The global oleochemicals market was valued at USD 38.64 billion in 2024 and is estimated to reach USD 40.65 billion in 2025 [5]. Estimates indicate that this increasing trend will continue in the next years, with long-term projections surpassing USD 87.62 billion by 2037 at a compound annual growth rate (CAGR) above 6.5%. This continuous expansion underscores the strong and growing demand for renewable feedstocks in diverse industrial sectors such as food, pharmaceuticals, and personal care [6]. However, from a sustainability and socioeconomic point of view, the oleochemical production from edible oils generates great concern [7]. This occurs because edible oil production requires arable lands, substantial amounts of water and fertilizers, competing with food production [8]. To overcome these drawbacks, it is pivotal to produce alternative oil sources, such as yeast oil. Importantly, the yeast oil production does not depend on geographical conditions, requires less space for production, and has higher lipid yields compared to plant oil production [9]. Moreover, oleaginous yeasts display several advantages, such as higher growth rates, the ability to reach high lipid contents, a similar fatty acid composition to vegetable oils, high volumetric productivity, and broad metabolic diversity. Various species of non-conventional oleaginous yeasts can grow on a wide range of sugars derived from different agroindustrial substrates, such as lignocellulosic biomasses, which include cellulose and hemicellulose, and cheese and ricotta whey, which provide lactose, representing a low-cost carbon source for oil production by these species [10].

Oleaginous yeasts, which accumulate at least 20% of their dry weight in lipids, are capable of producing high amounts of lipids [9]. This takes place when these yeasts are cultivated under high C:N rations, as the nitrogen depletion triggers the activity of adenosine monophosphate deaminase, which converts adenosine monophosphate (AMP) into inosine monophosphate and ammonia to obtain nitrogen. This reduction in cellular AMP content, which is an allosteric activator of the enzyme isocitrate dehydrogenase, leads to citrate accumulation in mitochondria. Subsequently, citrate is transported to the cytosol and cleaved by ATP-citrate lyase (ACL) into acetyl-CoA and oxaloacetate. Acetyl-CoA is carboxylase to malonyl-CoA by acetil-CoA carboxylase (ACC), which in turn is directed towards de novo fatty acid synthesis by the fatty acid synthase (FAS) complex. The fatty acids synthesized are subsequently channeled to the endoplasmic reticulum for the production of triacylglycerols (TAGs) and other lipids. Besides nitrogen, other nutritional limitations such as phosphorus, iron, and magnesium also stimulate the oleaginous phenotype in some organisms [11].

*Papiliotrema laurentii* is a non-conventional oleaginous yeast belonging to the Basidiomycota phylum, exhibiting dimorphism, capsule synthesis capability, and a lack of motility [12]. Additionally, *P. laurentii* can assimilate various carbon sources, such as glucose, xylose, arabinose, cellobiose, mannose, galactose, rhamnose, sucrose, lactose, and galacturonic acid [13]. Moreover, it accumulates lipids quickly than other native xylose-asimilating oleaginous yeasts such as *Rhodotorula toruloids* from culture media with xylose as carbon source, reaching maximum production within 48 h of cultivation [14]. In a culture medium containing xylose as the carbon source and optimized conditions (30 °C; 300 rpm; OD600 = 0.8, and pH = 7), *P. laurentii* achieved 63.5% of its dry weight in lipids [14]. Nevertheless, both biomass and lipid titers were lower compared to other studies due to the limited growth under these conditions. The cultivation of *P. laurentii* is generally carried out so far in culture media commonly used for oleaginous yeast in order to favor lipid accumulation. Due to limited knowledge about its physiological characteristics, designing factorial experiments becomes challenging, resulting in a more intuitive experimental design. Overall, most of the optimization studies focus on statistical designs such as Plackett–Burman, Box-Benken, and Taguchi methods to optimize microorganism cultivation [15–17]. Indeed, there are no systematic studies combining metabolic modelling and statistical design to optimizing biomass and lipid production in *P. laurentii.* This study is the first to systematically address these objectives by integrating a genome-scale metabolic model (GEM)-guided flux balance analysis (FBA) with Central Composite Rotational Design (CCRD)*.*

The first genome-scale metabolic model of *P. laurentii* was recently published [18], providing a useful tool to better understand its physiology [19]. The analysis of metabolic fluxes allows for gaining insights about the responses of a microorganism to environmental variations, identifying constraints in metabolic pathways that limit the production of specific compounds, evaluating levels of metabolic control, and establishing predictive models relevant to the development of improvement and processing strategies [20]. Flux balance analysis (FBA) uses linear programming to predict the distribution of fluxes by imposing constraints to maximize or minimize a specific objective. However, to apply this approach to study metabolic fluxes, it is necessary to construct a stoichiometric matrix representing the reactions and metabolites present in an organism. GEMs are based on the stoichiometric matrix, in silico reconstructions that mathematically represent the metabolism of an organism based on genomic annotation and cellular metabolic capabilities, linking genes, proteins, and reactions, thus relating genotype to phenotype [21].

The oxygen level of the culture medium also affects the growth, lipid content and fatty acid profile of oleaginous yeasts [22]. As such, assessing the volumetric oxygen mass transfer coefficient (*k*_*L*_*a*) is pivotal to designing and scaling up the lipid production by yeasts [23]. The optimization of biomass and metabolite production by microorganisms typically relies on factorial experimental designs involving a large number of experimental units [15]. This approach is still more challenging when optimizing biomass or metabolite production by non-model microorganisms, as their physiological features are not widely understood, making the experimental design more intuitive. This study presents a methodology to rationally guide the factorial experimental design for evaluating the effect of nutritional sources on microbial biomass production. The first stage of this work focused on identifying carbon and nitrogen sources that favor the growth of *P. laurentii* using FBA. Predictions obtained via FBA guided the factorial design, allowing the assessment of the effects of different combinations of carbon and nitrogen sources on the growth rate of *P. laurentii*. The highest biomass yields were recorded in culture media containing lactose and urea as carbon and nitrogen sources, respectively. Since these sources are low-cost, they were used in the next steps. Next, we evaluated the effects of the C:N ratio, *k*_*L*_*a*, and initial inoculum on biomass production and lipid titer. Lastly, the conditions that resulted in the highest lipid titer were applied to bioreactor cultivations, where the effect of increasing *k*_*L*_*a* values on biomass production and lipid titer was assessed. The adopted strategy herein was efficient, leading to significant biomass and lipid production increases by *P. laurentii*. Therefore, this work is the first to combine genome-scale metabolic modeling with factorial design and bioreactor optimization in *P. laurentii*, establishing a novel and integrative framework for process development in this yeast.

## Materials and methods

### Model curation and FBA

In the model curation step, unbalanced reactions were identified using a combined method. To identify unbalanced reactions, we employed the getElementalBalance function from the RAVEN 2.0 toolbox [25], a software suite for Matlab for genome-scale model reconstruction, flux analysis, and quality assessment, together with MEMOTE [26], a tool designed to evaluate the quality and consistency of GEMs. Reactions related to metabolite exchange and lipid structures added by SLIMEr were not considered. To correct the identified imbalances, the elemental composition of compounds within the reactions was checked against the MetaCyc and KEGG databases.

FBA simulations were conducted using the papla-GEM model, available from the GitHub repository (https://github.com/SysBioChalmers/papla-GEM), to predict flux distributions with biomass formation set as the objective function. FBA were conducted using RAVEN 2.0 toolbox and the Gurobi solver (version 9.1.1., Gurobi Optimization LLC, USA), a solver that uses mathematical optimization to calculate the answer to a problem, such as linear programming, to solve the FBA. Glucose uptake rates determined in both minimal and complex media under several cultivation conditions were used to provide the necessary constraints for the simulations [18]. To control the carbon source uptake rate, experimental data obtained during the exponential phase of a batch culture carried out in the minimal medium (Yeast Nitrogen Base, YNB) containing 5 g/L of glucose as the carbon source, at 30 ºC and an initial pH of 4.5 [18]. This data was used as the reference condition for the FBA. In this case, the glucose uptake rate was constrained to 5 mmol/gDWh, where DW corresponds to cell dry weight, as determined experimentally, while other carbon sources had their uptake rates adjusted to supply the same amount of carbon per unit of time. To identify the most favorable combination between carbon and nitrogen sources for the growth of *P. laurentii*, 10 different carbon sources (acetate, lactate, glycerol, glucose, xylose, arabinose, galactose, mannose, lactose, and sucrose), were combined with 25 nitrogen sources (ammonium sulfate as the standard inorganic source, urea, allantoin, ornithine, citrulline, and the 20 proteinogenic amino acids). Each combination was evaluated considering a specific uptake rate of 1 mmol/gDWh from the nitrogen source, a rate determined according to the range of fluxes predicted for ammonium consumption by Ventorim et al. [18]. The biomass yield is represented as the ratio between the biomass production flux and the carbon uptake rate.

### Microorganism and cultivation conditions

The *P. laurentii* UFV-1 strain used in this study belongs to the microbial culture collection of the Laboratory of Microbial Physiology at the Department of Microbiology, Universidade Federal de Viçosa. The yeast was stored at -80 °C in YP medium (10 g/L yeast extract, 20 g/L peptone) containing 50% (v/v) glycerol. This strain was isolated from a soil sample collected at the Serra dos Órgãos National Park [14].

First, *P. laurentii* UFV-1 was activated in a medium containing 10 g/L yeast extract, 20 g/L peptone, 20 g/L glucose, and 20 g/L agar (YPD-Agar) at 30 °C for 48 h. Then, a single colony was transferred to a 125 mL Erlenmeyer flask containing 25 mL of SS2 medium [27] with a modification in ammonium sulfate concentration. The SS2 medium consisted of 30 g/L glucose, 0.523 g/L ammonium sulfate, 0.5 g/L magnesium sulfate, 0.1 g/L calcium chloride, 0.1 g/L sodium chloride, and 0.1 g/L yeast extract. The pH of the culture medium was 4.6. The cultures were then incubated in a shaker at 30 °C and 200 rpm for approximately 19 h. The cultures were centrifuged at 4000 g for 10 min at 4 °C. The cell pellet was washed twice with water containing 0.85 g/L sodium chloride and resuspended in 5 mL of water with 0.85 g/L sodium chloride. The optical density at 600 nm (OD_600_) was then measured, and based on the value obtained, a specific volume of the cell suspension was transferred to new cultivation media in agreement with the experimental design, so that the initial OD_600_ of the cultures was approximately 0.1.

### FBA-guided experimental design to evaluate the nutrient effects on the biomass production by ***P. laurentii***

To evaluate the effect of the nutrients selected through FBA based on biomass yield calculation and the cost of the evaluated sources on biomass production, a triple factorial experiment (4 × 3x2) was conducted using a completely randomized design with two replicates per treatment. Statistical analyses were performed using the R software, version 4.1.1. The evaluated factors included: 4 carbon sources (lactose, sucrose, glucose and xylose), 3 concentrations for each carbon source (for glucose and xylose: 10, 20 and 30 g/L; for lactose and sucrose: 9.52, 19.04 and 28.56 g/L), and 2 nitrogen sources (urea and ammonium sulfate), with nitrogen concentrations adjusted to maintain a C:N ratio of 100:1 to induce the oleaginous phenotype (for ammonium sulfate: 0.143, 0.333 and 0.523 g/L, for urea: 0.064, 0.149 and 0.234 g/L). The cultivations were carried out at 30 ºC, 200 rpm for 48 h. The choice of carbon and nitrogen sources was based on the biomass yield predicted by FBA, as well as the availability of these substrates, taking the economic impact of using such sources into account. For the carbon sources, glucose and sucrose were included as sugars found in feedstocks employed first-generation biofuels such as sugar cane and corn. Their use in large-scale microbial oil production competes with the food industry [28]. On the other hand, xylose and lactose were selected as second-generation substrates chosen for their high availability from non-food sources. Glucose and xylose are the major components of lignocellulosic hydrolysates, derived from abundant agricultural residues, as straw and bagasse [29]. Lactose is a key component of cheese whey, a high-volume byproduct of the dairy industry. The utilization of these second-generation substrates is paramount for ensuring a sustainable supply of raw material [30]. For the nitrogen sources, urea and ammonium sulfate were selected as commercially available fertilizers [31]. Studies have shown that urea is a sustainable alternative to ammonium sulfate, often resulting in comparable or even improved growth and lipid accumulation [32, 33]. A Shapiro–Wilk test was conducted at a 5% significance level to verify the normality of residuals, and a Bartlett test at a 5% significance level was used to check the homogeneity of residual variances by treatment. These analyses were performed to assess the feasibility of using analysis of variance (ANOVA). Treatments were evaluated using ANOVA with Tukey’s test at a 5% significance level.

After completing the 4 × 3x2 factorial experiment and selecting the best combination of carbon and nitrogen sources (lactose and urea, respectively), a new 4 × 3 factorial experiment with two replicates per treatment was conducted as mentioned above. In this experiment, four concentrations of yeast extract (0.1, 0.5, 1.0, and 1.5 g/L) and three concentrations of the selected carbon source (12, 16, and 20 g/L of carbon) were evaluated. To maintain the same ratio of lactose and urea from the previous factorial experiment, the additional nitrogen content resulting from the increased yeast extract concentration was not considered in the calculation of the C:N ratio. This approach prioritized the best combination between the carbon and nitrogen concentrations, which as previously determined in the factorial experiment that led to the highest biomass production. The C:N ratio remained at 100:1 only when the yeast extract concentration was 0.1 g/L. The reference cultivation medium was a modified SS2 medium with glucose and ammonium sulfate: magnesium sulfate (0.5 g/L), sodium chloride (0.1 g/L), calcium chloride (0.1 g/L), yeast extract (0.1 g/L), ammonium sulfate (0.523 g/L), and glucose (30 g/L) [27].

### Biomass determination

Cellular biomass (g/L) was determined gravimetrically after 48 h of cultivation. The cultures were transferred to pre-dried, pre-weighed 50 mL conical centrifuge tubes. Subsequently, they were centrifuged at 1912 g for 10 min at 4 °C. The sedimented cells were then washed twice with water containing 0.85 g/L sodium chloride, and the supernatant was discarded. The sedimented cells in the conical centrifuge tubes were then frozen in liquid nitrogen and lyophilized. After this procedure, the samples were stored in a desiccator until the dry weight remained constant. In addition to biomass determination, OD_600_ and final biomass were measured after the 48-h cultivation period.

### Lipid production

About 50 mg of dry mass was transferred to 2 mL microtubes (MCT-200-C, Axygen, USA) for lipid extraction. Lipid extraction was performed following the method proposed by Bligh and Dyer [34], with modifications [14]. First, 1 mL of a methanol solution (2:1) and two tungsten beads were added to the tubes containing the biomass. The samples were then macerated using a ball mill (TissueLyserII, Qiagen N.V., Netherlands) programmed for 30 oscillations per second for 5 min. Afterward, samples were centrifuged at 13,201* g* for 10 min at 10 °C, and the supernatant was transferred to glass centrifuge tubes. This procedure was repeated twice. Then, 3 mL of chloroform was added to the glass centrifuge tubes containing the supernatants, and the mixture was homogenized with a pipette, followed by the addition of 2 mL of a 1% (w/v) NaCl solution to promote phase separation. The mixture was centrifuged at 1111 g for 20 min at 10 °C, and the organic phase was collected and stored in pre-weighed microtubes. The solvent was evaporated in a fume hood using a dry bath at 60 °C for 20 h, and the extracted lipids were dried in an oven at 60 °C until a constant mass was reached. The lipid content was expressed as a percentage.

### Optimization of growth conditions

Upon defining the culture medium composition that favors the biomass production by *P. laurentii* an optimization of cultivation conditions was performed, evaluating initial OD_600_, C:N ratio, and aeration (represented by *k*_*L*_*a*). A Central Composite Rotational Design (CCRD) was applied to evaluate the impact of each factor on biomass production and lipid titer and determine the best culture condition in the flask to increase lipid titer. The experimental ranges for each factor were as follows: initial OD_600_ (0.1, 0.24, 0.45, 0.65, 0.8), C:N ratio (60, 68, 80, 91, 100), and *k*_*L*_*a* (h^−1^) (11.24, 12.32, 16.31, 21.96, 28.41). The cultivations were carried out at pH 7.3, 30 °C, and 200 rpm for 48 h. The effects of each variable were evaluated by ANOVA. Information on variance analysis and CCRD execution are found in the supplementary material.

### Determination of the ***k***_***L***_***a***

The *k*_*L*_*a* was estimated in flasks of 125 mL from cultivation parameters and incubator characteristics. To estimate *k*_*La*_ in seconds, the following equation was applied:

1 $$k_{L} a\left( {\mathrm{s}} \right) \, = { 6},{67}.{1}0^{{ - {6}}} {\text{x R}}^{{{1},{16}}} {\text{x V}}^{{ - 0,{83}}} {\text{x Da}}^{{0,{38}}} {\text{x Df}}^{{{1},{92}}}$$

where R is the stirring frequency in RPM (R); V is the working volume in the flask in mL; Da is the stirring diameter in centimeters; and Df is the base diameter of the flask in centimeters [35]. For each cultivation, the fixed parameters were: agitation speed (R) of 200 rpm, impeller diameter (Da) of 2 cm, and flask diameter (Df) of 4,5 cm.

The *k*_*L*_*a* for the bioreactor cultures was determined using the gassing-out method. To reduce the dissolved oxygen in the culture medium to zero, nitrogen gas was bubbled until a constant reading was detected on the oxygen sensor, which was considered as 0% oxygen. Afterward, oxygen flow into the medium was reestablished at 0.5 Volumes of air/gas per Volume of Medium per Minute (VVM), and once the oxygen sensor stabilized, the point was defined as 100%, that is, the saturation of the medium with oxygen under a specific condition. For *k*_*L*_*a* determination, the linear region of the oxygen transfer rate after its complete removal from the culture medium was used to calculate the *k*_*L*_*a* according to the following equation [36]:

2 $$K_{L} a\left( {t2 - t1} \right) \, = ln(\left( {Cs - CL1)/(Cs - CL2)} \right)$$

where Cs is the oxygen saturation concentration; CL1 and CL2 are the oxygen concentration at the beginning of the experiment and at times t1 and t2, respectively.

### Bioreactor cultivations

The cultivations were performed in a 1.3 L bioreactor (BioFlo®/CelliGen® 115, Eppendorf) with 900 mL of culture medium. The conditions of cultivation were defined in agreement with the lipid titers recorded from the CCRD. The impact of *k*_*L*_*a* in the system was evaluated at 30 ºC, pH 7.3, by 48 h, with three different values (28.41, 49.42, and 103.15 h^−1^). Antifoam 0.01% (V/V) was added to the culture medium. Experiments were carried out in duplicate, and the results were evaluated by ANOVA with the Tukey post-hoc test at 5% significance level. This method involved varying agitation speeds (100 to 800 rpm) while maintaining a constant airflow rate of 0.5 VVM (see Supplementary materials). The *k*_*L*_*a* data obtained were correlated with the respective agitation rates, allowing the construction of a curve to represent the data points and derive an equation relating agitation speed to *k*_*L*_*a*. This equation predicted the agitation speed required to achieve a specific *k*_*L*_*a* within the tested range. Thus, the bioreactor agitation corresponding to the *k*_*L*_*a* value calculated from flask cultivation, optimized for lipid production via the CCRD, was established. Additional *k*_*L*_*a* values were determined based on 300 and 400 rpm agitation speeds. By using the gassing-out method, we observed that increasing the agitation beyond 400 rpm did not result in further *k*_*L*_*a* enhancement for the culture medium developed in the current study, maintaining a volume of the medium: volume of the flasks (MV:FV) ratio of 0.69.

## Results and discussion

### Biomass yield prediction for different carbon and nitrogen source combinations

We identified 411 unbalanced reactions during the model curation; 93 exclusive reactions were found by the *getElementalBalance* function from RAVEN 2.0, and 44 by MEMOTE (see Supplementary materials). To balance the reactions, the MetaCyc and KEGG databases were used to check the elemental composition of the compounds belonging to the reactions. A total of 409 reactions (99.5%) were successfully balanced. The remaining reactions involved compounds with undefined structures, limiting further correction. Balancing these reactions contributed to accurate mass conservation, enhancing the reliability of flux balance analyses and other model-based metabolic predictions. Reactions related to the lipid metabolism added to papla-GEM by using SLIMEr, which is a formalism for correctly modeling lipid metabolism in genome-scale metabolic models [37], were not evaluated in this curation process due to the use of abstract or generic lipid species without defined molecular structures. It is noteworthy that these reactions often represent lipid classes or aggregated processes without precise elemental composition; thus, it is impossible to guarantee mass and charge balance.

**Fig. 1:**
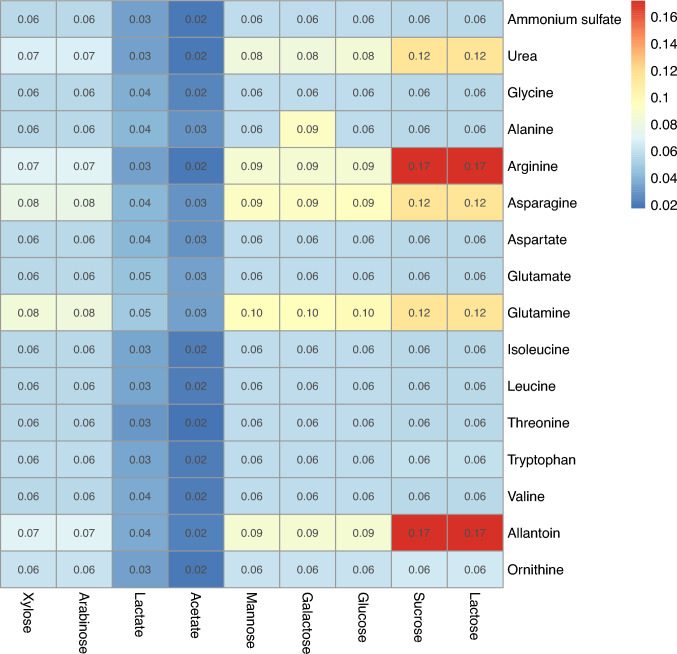
Heatmap depicting the biomass yield of *P. laurentii* predicted by FBA under varying carbon and nitrogen source combinations. Red indicates higher yield; blue indicates lower yield

Lactose and sucrose were selected herein as carbon sources that favor the biomass production by *P. laurentii.* The predicted biomass yield, calculated as the ratio between the biomass formation flux and the limiting nutrient uptake rate, suggests that the yeast may exhibit higher metabolic efficiency in nutrient utilization within the combinations evaluated in the simulations [38, 39]. Biomass yield is a key metric for the optimization of culture media and for the selection of nutrient combinations in FBA simulations, often serving as an indicator of high-yield metabolic pathways [40, 41]. Highest biomass yields were predicted mainly when these sugars were combined with allantoin and arginine as nitrogen sources. Likewise allantoin and arginine, glutamine, asparagine, and urea displayed high biomass yields when combined with other carbon sources (Fig. 1). Importantly, we did not detect flux directed towards the growth of *P. laurentii* when citrulline, cysteine, histidine, lysine, methionine, phenylalanine, proline, serine, and tyrosine were evaluated as nitrogen sources, regardless of the carbon sources considered herein. For this reason, these nitrogen sources were not depicted in Fig. 1. Even though the biomass yield predicted for allantoin and arginine was higher than that of other nitrogen sources, their industrial use is considered economically unfeasible. Allantoin is primarily obtained via chemical synthesis or extraction from plant sources, both of which are costly and present low yields; as such, it is not reasonable to include it in large-scale processes [42, 43]. In the case of arginine, techno-economic analyses show that its addition in culture media significantly increase the nutrient cost in biomass-based systems; therefore, its use is uncompetitive compared to other nitrogen sources such as urea [44–46]. Although glutamine and asparagine also stood out as promising nitrogen sources, it is not feasible to include them in large-scale bioprocesses. As such, the nutrient selection in this study was based not only on flux to biomass yield, but also on the industrial availability and cost of the carbon and nitrogen sources, as the fermentation media can represent up to 80% of the total cost of a bioprocess [47, 48]. Since amino acids are nitrogen sources that generally improve biomass production, simulations with these nitrogen sources were performed to compare the biomass yields between them and lower-cost nitrogen sources such as urea.

In this sense, we decided to select glucose as representative of hexoses, which is found in the cellulosic fraction of lignocellulosic biomasses, and xylose as representative of pentoses, as it is the most abundant sugar found in the hemicellulosic fraction of lignocellulosic biomasses. These biomasses are abundant and low-cost agroindustrial byproducts used as raw materials in biorefineries [49, 50]. With regard to disaccharides, we selected lactose, which is a constituent of whey, a byproduct of the dairy industry, and sucrose, which is found in large quantities in sugarcane molasses [24, 51, 52]. Taking the nitrogen sources into account, ammonium sulfate, a common and cheap nitrogen source, was selected. Urea, which is a low-cost nitrogen source found in fertilizers and various residues such as municipal wastewater, wood industry residues, and agricultural residues, was also selected [53, 54]. Therefore, although the optimal combinations identified in the FBA pointed to lactose or sucrose paired with arginine or allantoin, the pairing of lactose or sucrose with urea, from a biotechnological point of view, emerges as the most promising alternative. In summary, based on FBA and nutrient source feasibility, the nitrogen sources selected for the factorial experiment were urea and ammonium sulfate as the organic and inorganic nitrogen sources, respectively. Regarding the carbon sources, glucose (reference), xylose, lactose, and sucrose were chosen for further steps.

### Optimization of carbon and nitrogen sources for biomass and lipid production by *Papiliotrema laurentii*

In the first optimization step, *P. laurentii* was cultivated at concentrations of 4, 8, and 12 g/L of carbon for all carbon sources, maintaining a carbon-to-nitrogen (C:N) ratio of 100:1 to favor lipid with according to Vieira et al. [14]. We observed significant double interactions between the variables carbon concentration and nitrogen source, carbon concentration and carbon source, and carbon and nitrogen source, with a coefficient of variation (CV) of 7.59% (Table 1). Regarding the interaction between carbon concentration and nitrogen source, we verified that the increase in the carbon concentration affected the biomass production for both nitrogen sources. Moreover, we did not observe a statistical difference between ammonium sulfate and urea at a carbon concentration of 4 g/L in final biomass; on the other hand, urea favored biomass production at carbon concentrations of 8 and 12 g/L. These results are consistent with the FBA predictions, demonstrating that lactose and urea were, in fact, the best sources of carbon and nitrogen, respectively, among the sources evaluated, and that the combination of these sources provided the highest final biomass concentration, underscoring the suitability of GEMs to identify in silico nutrient sources for developing culture media.

**Table 1:** Final biomass (g/L) of *P. laurentii* cultivated in different carbon and nitrogen sources: evaluation of dual interactions: carbon concentration-nitrogen sources, carbon sources-carbon concentration, and carbon sources-nitrogen sources

Interaction: carbon concentration and nitrogen source				
	Carbon concentration (g/L)			
Nitrogen sources	4	8	12	Regression equation
Ammonium sulfate	1.26^a^	1.41^b^	1.37^b^	Y = 0.0137x R^2^ = 0.582
Urea	1.34^a^	1.60^a^	1.74^a^	Y = 1.168 + 0.0491x R^2^ = 0.969
**Interaction: carbon source and carbon concentration**				
	**Carbon concentration (g/L)**			
**Carbon sources**	**4**	**8**	**12**	**Regression equation**
Glucose	1.21^b^	1.40^b^	1.32^c^	###
Xylose	1.19^b^	1.32^b^	1.36^bc^	###
Sucrose	1.32^ab^	1.45^b^	1.54^b^	Y = 1.221 + 0.0273x R^2^ = 0.992
Lactose	1.47^a^	1.86^a^	1.99^a^	Y = 1.262 + 0.0641 R^2^ = 0.930
**Interaction: carbon source and nitrogen source**				
	**Carbon sources**			
**Nitrogen sources**	**Glucose**	**Xylose**	**Sucrose**	**Lactose**
Ammonium sulfate	1.26^aB^	1.23^aB^	1.39^aAB^	1.51^bA^
Urea	1.37^aB^	1.35^aB^	1.49^aB^	2.04^aA^

**For the carbon concentration-nitrogen sources interaction**, data are presented as the mean of two replicates. Different letters indicate significant differences in the Tukey test (p < 0.05) between the nitrogen sources, observing the columns, while the regression equations refer to the effect of carbon concentration on the biomass concentration. p-value of the interaction: 0.0048.

**For the carbon sources-carbon concentration interaction**, data are presented as the mean of two replicates; different letters indicate significant differences in the Tukey test (p < 0.05) between the carbon sources, observing the columns. The regression equation refers to the effect of carbon concentration on biomass concentration, while the symbols ### represent the absence of the effect of carbon concentration on the response variable. p-value of the interaction: 0.0236.

**For the carbon sources-nitrogen sources interaction**, data are presented as the mean of two replicates. Different letters indicate significant differences in the Tukey test (p < 0.05). Lowercase letters compare means across observed nitrogen sources in columns, while uppercase letters compare means across observed carbon sources in rows. p-value of interaction: 1^e−04^

We also observed a positive effect of the carbon concentration on the biomass production (*p* < 0.05) in the presence of ammonium sulfate. Indeed, carbon concentrations above 4 g/L led to the highest biomass concentrations. However, it was not possible to establish a representative regression model between biomass and carbon concentration. Therefore, we were unable to determine the carbon concentration that maximizes the biomass production. In contrast to ammonium sulfate, we observed a linear and increasing trend of biomass production in cultivations with either sucrose or lactose in the presence of urea. This suggests that the biomass production might be higher at carbon concentrations superior to 12 g/L.

Upon analyzing the interaction between carbon source and carbon concentration (Table 1), we observed that for all evaluated concentrations, cultures grown in media containing lactose yielded higher biomass production, except at 4 g/L of carbon, where biomass concentrations were similar between lactose and sucrose. For these two sugars, the effect of carbon concentration was linear and increasing (*p* < 0.05), which was not observed in cultures with glucose or xylose. Notably, within the same range of carbon concentration variation, lactose favored biomass production more than twofold as much as sucrose. Based on these findings, the combination of lactose and urea as carbon and nitrogen sources, respectively, appears to be the most suitable.

Regarding the interaction between carbon and nitrogen sources, we observed that the final biomasses were similar for both ammonium sulfate and urea, except when lactose was used as the carbon source (Table 1). For lactose, urea significantly enhanced biomass production. Overall, the biomasses achieved by *P. laurentii* in fermentation media containing lactose were higher for both ammonium sulfate and urea. Moreover, the combinations of sucrose and ammonium sulfate and lactose with ammonium sulfate did not significantly differ. These results are remarkably in agreement with the FBA, as the biomass yield predicted on urea was higher than on ammonium sulfate. Furthermore, the biomass production in lactose was higher than in other carbon sources, regardless of the nitrogen source tested. Taken together, these results point out that the combination of lactose and urea is suitable for biomass production by *P. laurentii*. Indeed, there was an increase of approximately 63% in biomass concentration compared to the reference condition, which corresponds to the cultivation in a modified SS2 medium with glucose and ammonium sulfate: magnesium sulfate (0.5 g/L), sodium chloride (0.1 g/L), calcium chloride (0.1 g/L), yeast extract (0.1 g/L), ammonium sulfate (0.523 g/L), and glucose (30 g/L) [27] (Table 4).

*P. laurentii* exhibited the oleaginous phenotype, that is, achieved at least 20% of its biomass consisting of lipids in all conditions evaluated (Table 2). Regarding the lipid titer, we observed a significant three-way interaction (*p* < 0.05), with a coefficient of variation (CV) of 10.32% (Table 3). The nitrogen source did not influence the lipid production by *P. laurentii* in cultivations containing xylose or sucrose; otherwise, the nitrogen source affected the lipid production when glucose was the carbon source. We obtained the highest lipid titer at a concentration of 12 g/L of carbon when lactose and urea were the carbon and nitrogen sources, respectively. Moreover, we observed that the urea favored lipid production at concentrations of 8 and 12 g/L of carbon. It should be noted that the lipid titers recorded in these lactose concentrations were higher than those achieved with other carbon sources (Table 3).

**Table 2:** Average lipid contents (%) of *P. laurentii* cultivated in different combinations of carbon and nitrogen sources and evaluation of the triple interaction

Lipid content (%)						
**Carbon concentration (g/L)**						
	4		8		12	
	Ammonium sulfate	Urea	Ammonium sulfate	Urea	Ammonium sulfate	Urea
**Glucose**	43.06^aA^	35.29^bA^	39.35^aA^	37.06^aA^	43.34^aA^	32.03^bA^
**Xylose**	30.12^aB^	34.04^aA^	39.12^aA^	31.06^bA^	37.42^aAB^	30.82^bA^
**Sucrose**	36.89^aA^	37.27^aA^	38.79^aA^	36.51^aA^	38.55^aAB^	37.0^aA^
**Lactose**	38.57^aA^	35.11^aA^	36.27^aA^	34.25^aA^	35.99^aB^	32.28^aA^

Data are presented as means of two replicates. Different letters indicate significant differences in the Tukey test (p < 0.5). Lowercase letters compare the means between the nitrogen sources observed in the rows, at a given carbon source and concentration. In contrast, uppercase letters compare the means between the carbon sources observed in the columns, at a given nitrogen source and concentration. The CV of the data presented a value of 6.58%. p-value of the triple interaction: 0.0281

**Table 3:** Mean lipid titers (g/L) of *P. laurentii* grown in different combinations of carbon and nitrogen sources: evaluation of triple interaction

Lipid titer (g/L)						
Carbon concentration (g/L)						
	4		8		12	
	Ammonium sulfate	Urea	Ammonium sulfate	Urea	Ammonium sulfate	Urea
Glucose	0.565^aA^	0.394^bB^	0.52^aA^	0.552^aB^	0.496^aA^	0.481^aBC^
Xylose	0.336^aB^	0.432^aAB^	0.501^aA^	0.419^aB^	0.486^aA^	0.437^aC^
Sucrose	0.471^aAB^	0.557^aA^	0.572^aA^	0.503^aB^	0.552^aA^	0.613^aB^
Lactose	0.518^aA^	0.566^aA^	0.572^bA^	0.731^aA^	0.577^bA^	0.766^aA^

Data are presented as means of two replicates. Different letters indicate significant differences in the Tukey test (p < 0.5). Lowercase letters compare the means between the nitrogen sources observed in the rows, at a given carbon source and concentration, while uppercase letters compare the means between the carbon sources observed in the columns, at a given nitrogen source and concentration. The CV of the data presented a value of 10.32%. p-value of the triple interaction: 0.0121

With regard to carbon concentrations, the combination of lactose and urea displayed a linear behavior (Y = 28.2547 + 0.9125x; R^2^ = 0.876; *p* < 0.05), indicating that lipid titer increased along with the carbon concentration. Additionally, a positive effect on lipid titer was recorded for the combination of xylose and ammonium sulfate. Xylose concentrations above 4 g/L were required to achieve the higher lipid titers. However, it was not possible to fit a representative regression model for lipid titer and carbon concentration supplied by xylose. Thus, it is not possible to conclude if a concentration of 12 g/L of carbon is necessary.

For the combination of glucose and urea, there was no significant linear effect with the increase in the carbon concentration. Nevertheless, we noted an increase in lipid titer when carbon concentration increased from 4 to 8 g/L. Otherwise, we did not observe an increase when the lactose ranged from 8 to 12 g/L. For other combinations of carbon and nitrogen sources, the carbon source concentration did not significantly affect the average lipid titers (Table 2).

Based on these results, we point out that the combination of lactose and urea as carbon and nitrogen sources, respectively, can be used for lipid production by *P. laurentii*. This combination enabled a significantly higher biomass concentration, with an increase of approximately 63% compared to the reference condition, which consisted of the modified SS2 medium by Tanimura et al. [27] using glucose at 12 g/L of carbon (glucose at 30 g/L) and ammonium sulfate at 0.523 g/L. Additionally, a 54% increase in lipid titer was achieved compared to the reference condition (Table 4). The results demonstrated a linear correlation between increasing lactose concentration and biomass production. Since the lipid extraction was performed at 48 h for all cultures, the volumetric lipid productivity followed the same profile of lipid titer.

**Table 4:** Fermentation parameters of *P. laurentii* grown under standard and optimized conditions

Fermentation parameters				
Cultivation Condition	Biomass (g/L)	Lipid content (%)	Lipid titer (g/L)	Volumetric productivity (mg/L h^−1^)
Standard Condition: SS2; A.S (0.523 g/L); Glucose (30 g/L); Y.E (0.1 g/L)	1.26	43.34	0.496	10.33
Optimization 1: SS2; Ur (0.234 g/L); Lactose (28.56 g/L); Y.E (0.1 g/L)	2.04	32.28	0.766	15.96
Optimization 2: SS2; Ur (0.234 g/L); Lactose (28.56 g/L); Y.E (0.5 g/L)	7.52	40.17	2.99	62.30
Optimization CCRD, Biomass: *k*_*L*_*a* (28.41/h); C:N ratio (63.6); initial OD (0.8 SS2; Ur (0.234 g/L); Lactose (28.56 g/L); Y.E (0.5 g/L)	10.82	19.27	2.08	43.33
Optimization CCRD, Lipid titer: *k*_*L*_*a* (28.41/h); C:N ratio (100); initial OD (0.1) SS2; Ur (0.234 g/L); Lactose (28.56 g/L); Y.E (0.5 g/L)	8.9	39	3.48	72.29
Optimization *k*_*L*_*a* bioreactor: SS2; Ur (0.234 g/L); Lactose (28.56 g/L); Y.E (0.5 g/L) *k*_*L*_*a* (49.42/h); C:N ratio (100); initial OD (0.1)	9.97	46	4.54	94.58

SS2, A.S, Ur, and Y.E correspond to the culture medium, ammonium sulfate, urea, and yeast extract, respectively. The cultures were carried out at 30 ºC with shaking at 200 rpm for 48 h

### Optimization of carbon and yeast extract concentration for biomass and lipid production

Since lactose and urea stood out as a suitable combination for both biomass and lipid production by *P. laurentii*, a new 4 × 3 factorial experiment was carried out. This experiment varied both lactose concentration (28.56, 38.08, and 47.6 g/L) and yeast extract concentration (0.1, 0.5, 1, and 1.5 g/L). The effect of the yeast extract concentration aimed to investigate whether higher levels of additional nutrients previously unidentified in FBA simulations could also enhance biomass production. This evaluation was based on the fact that additional nutritional requirements could stimulate biomass production by *P. laurentii*, a yeast isolated from soil, a nutrient-rich and diverse environment [55]. Yeast extract is a complex substrate containing a wide array of compounds, including proteins, amino acids, glycans, nucleotides, vitamins, and minerals. This composition provides a broad spectrum of nutrients potentially essential for the growth of specific microorganisms [56, 57]. Yeast extract is also a common component in various culture media [58], making it an important variable in terms of biomass production.

**Fig. 2:**
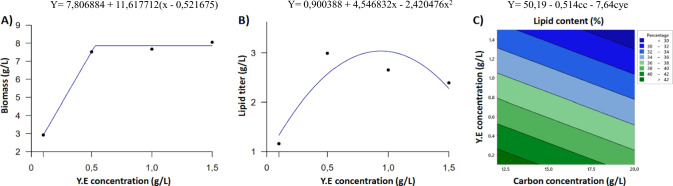
Effects of yeast extract on biomass concentration and lipid production. Y.E concentration represents the concentration of yeast extract. **A** Biomass in g/L. The statistics of the model were: *R*^2^ = 0.97; CV = 5.75%; p-value = 1.515 ^e−10^. **B** Lipid titer in g/L. The statistics of the model were: *R*^2^ = 0.69; CV = 17.88%; p-value = 1.441^e−06^. **C** Contour plot of the response surface model: Lipid content in g/L. The statistics of the model were: R^2^ = 0.60; CV = 9,84%; p-value = 2.704^e−05^. In the model, cc represents the carbon concentration supplied by lactose in g/L, while cye represents the yeast extract concentration in g/L. The axis representing yeast extract concentration shows a greater number of intersection points. The color scale used indicates the lipid content as a percentage, with dark green corresponding to a higher lipid content, while dark blue represents a lower lipid content

**Fig. 3:**
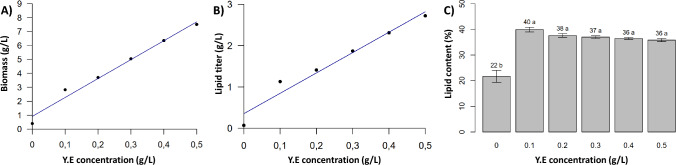
Effect of yeast extract at concentrations from 0 to 0.5 g/L. Y.E concentration represents the concentration of yeast extract. **A** Effect on biomass in g/L. The statistics of the model were: *R*^2^ = 0.98 CV = 8.79% p-value = 1.205e-09. **B** Effect on lipid titer in g/L. The statistics of the model were: R^2^ = 0.95 CV = 12.25% p-value = 3.48e-08. **C** Effect on lipid content (%)

We verified the significant effects of the yeast extract concentration on the biomass concentration and lipid titer (Figs. 2A and B). For lipid content, both yeast extract and carbon concentrations had significant effects (Fig. 2C). The best-fitting model for the biomass response was a linear plateau model (Fig. 2A). We verified that the biomass increased from yeast extract concentrations ranging from 0.1 to 0.5 g/L, resulting in a biomass concentration of 7.52 g/L at a concentration of 0,5 g/L (Fig. 3A). This biomass represents a substantial increase of 497% (Table 4) compared to the reference condition, which consisted of magnesium sulfate 0.5 g/L; sodium chloride 0.1 g/L; calcium chloride 0.1 g/L; yeast extract 0.1 g/L; ammonium sulfate 0,523 g/L; glucose 30 g/L. The highest lipid titer was obtained at 0.5 g/L of yeast extract, reaching 2.99 g/L of lipids (Fig. 2B). It should be noted that this lipid titer represented a significant increase of 503% (Table 4), also compared to the reference condition aforementioned. The response surface model analysis indicated that both lipid titer and lipid content decrease simultaneously with increases in yeast extract concentrations (Figs. 2B and C); however, the reduction in lipid content is more sensitive to the increase in yeast extract concentration, which is well evidenced in the contour plot (Fig. 2C). These results are likely associated with the higher nitrogen availability in the presence of elevated yeast extract concentrations. It is noteworthy that nitrogen-limiting conditions are pivotal to induce the adenosine monophosphate deaminase, leading to the reduction of AMP concentration, which is an allosteric activator of the isocitrate dehydrogenase, causing the reduction of its activity and citrate accumulation in mitochondria. Afterwards, citrate is transported to the cytosol where it is converted to acetyl-CoA by ATP-citrate lyase.

Since the maximum biomass production and maximum lipid titer were recorded at 0.5 g/L of yeast extract, we evaluated the effect of the yeast extract concentration on both biomass production and lipid titer within the range of 0 to 0.5 g/L. We verified a linear relationship for both response variables, underscoring the relevance of increasing yeast extract concentration up to 0.5 g/L for enhancing biomass production and lipid titer (Fig. 3). Moreover, we observed that the addition of yeast extract increased the lipid content; however, there were no statistically significant differences among the yeast extract concentrations evaluated (Fig. 3C).

Increasing the yeast extract concentration up to 0.5 g/L improved the biomass formation (Fig. 3A), corresponding to an increase of 370% compared to the best combination of the 4 × 3 × 2 factorial design (Table 4). However, yeast extract concentrations superior to 0.5 g/L reduced the lipid titers (Fig. 2B). These results underscore the importance of adding yeast extract for the *P. laurentii* growth, being 0.5 g/L the optimal concentration. Taken together, our results indicate that the modified SS2 medium of Tanimura et al. [27], containing lactose as the carbon source at 28.56 g/L, urea as the nitrogen source at 0.234 g/L, and yeast extract at 0.5 g/L, favors both biomass and lipid titers. Although both biomass and lipid production by *P. laurentii* were lower than those recorded for other yeasts, the volumetric lipid productivity was superior or comparable to values reported for other oleaginous yeasts [59, 60] (Table 4).

### Optimization of the cultivation conditions to increase both biomass and lipid production by *Papiliotrema laurentii*

**Fig. 4:**
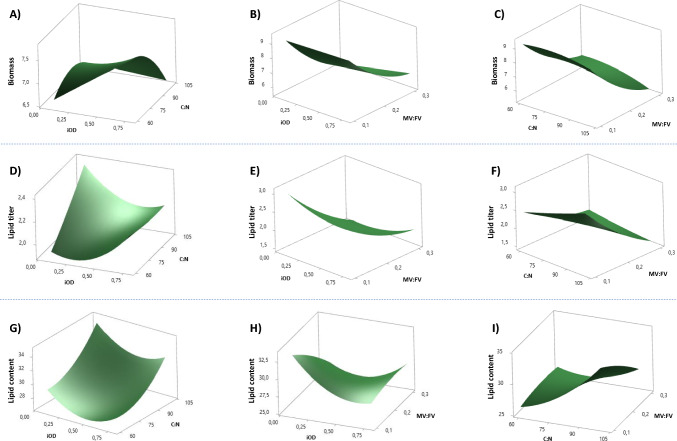
Response surface plots generated by a central composite design (CCD) analysis. Panels A-C visualize the interactive effects of initial optical density (iOD), C:N ratio, and *k*_*L*_*a* (represented by the MV:MF ratio) on biomass production (g/L). Panels D-F show the interactive effects of these same variables on lipid titer (g/L). Panels G-I display the interactive effects of these variables on lipid content (%)

The optimization of other important factors, such as initial inoculum size and aeration, may further enhance the biomass and lipid production by *P. laurentii*. To address this issue, a CCRD was performed to evaluate the effect of the variables *k*_*L*_*a* (represented by the MV:FV ratio), initial OD_600_, and the C:N ratio on biomass production. We observed that all variables significantly influenced biomass production. An increase in the inoculum size and *k*_*L*_*a* positively impacted biomass production; otherwise, an increase in the C:N ratio negatively affected this parameter due to nitrogen-limiting conditions (Fig. 4). The *k*_*L*_*a* was the main limiting factor for biomass production, underscoring the need for a high oxygen availability in the culture medium. Regarding the lipid titer, only *k*_*L*_*a* showed a significant effect, displaying a direct proportional relationship between increased *k*_*L*_*a* and lipid titer enhancement. Moreover, we observed a limited variation in the oleaginous phenotype across the tested C:N ratios. Although the C:N ratio influences the lipid content, its effect on lipid titer was less pronounced.

**Fig. 5:**
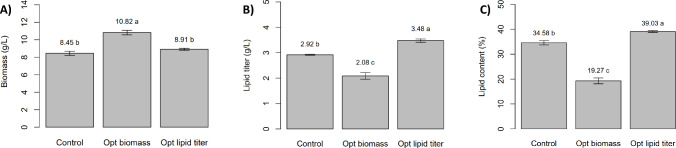
Optimizations by CCRD: This figure shows the effect of the conditions predicted by CCRD to optimize biomass and lipid titer production by the yeast *P. laurentii*. **A** Biomass in g/L at. **B** Lipid titer in g/L. **C** Lipid content in %

To validate the model generated by the CCRD, the predicted optimal conditions for biomass and lipid titer production were evaluated. It should be noted that the optimized conditions were compared to the reference condition (control), which was carried out under the following conditions: initial OD_600_ = 0.1, C:N = 75:1, *k*_*L*_*a* = 15.73 h^−1^ (Fig. 5), achieving 8.45 g/L of biomass and 2.92 g/L of lipid titer. For biomass production, the optimal conditions were: an initial OD_600_ of 0.8, a C:N ratio of 63:1, and *k*_*L*_*a* of 28.41 h^−1^. Regarding the lipid titer, the predicted optimal conditions were an initial OD_600_ of 0.1, a C:N ratio of 100:1, and *k*_*L*_*a* of 28.41 h^−1^. The predicted values for these conditions were 9.68 g/L for biomass and 3.47 g/L for lipid titer. Consistent with the predictions, the experimental validations were 10.82 g/L and 3.48 g/L of biomass and lipid titer, respectively, demonstrating the robustness and representativeness of the CCRD model (Fig. 5). As shown in Fig. 5C, when prioritizing cultivation conditions for biomass production, the lipid content was significantly lower compared to both the control and the lipid titer optimization condition. This result is likely related to the lower C:N ratio used for biomass optimization. In this ratio, nitrogen was not limiting as in the C:N ratio used for optimizing the lipid production. It is important to point out that nitrogen-limiting conditions are pivotal for the oleaginous phenotype in yeasts. Nitrogen limitation leads to the reduction of the isocitrate dehydrogenase activity, which in turn decreases the accumulation and subsequent excretion of citrate from the mitochondria into the cytosol. As such, the production of acetyl-CoA by ATP citrate lyase, which is the building block for fatty acid synthesis, decreases [11].

The *k*_*L*_*a* was the main limiting factor for both biomass and lipid production, as neither plateau nor maximum points were achieved. Hence, additional experiments were conducted in a bioreactor to better evaluate the effects of the *k*_*L*_*a* on both biomass and lipid production. The initial cultivation conditions for these experiments were selected based on the CCRD results, which favored lipid titer, once the biotechnological interest is in the lipid profile of *P. laurentii* as a promising feedstock for biodiesel production [14].

### Bioreactor cultures

**Fig. 6:**
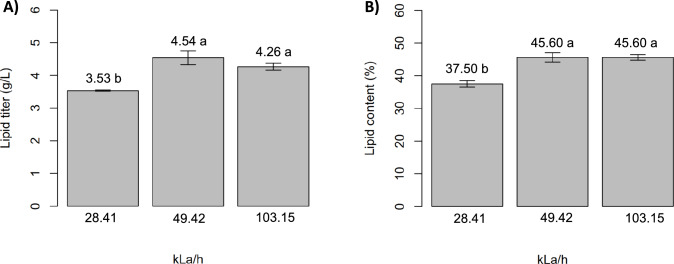
Impact of *k*_*L*_*a* on *P. laurentii* lipid production**.** **A** Lipid titer (g/L) at various *k*_*L*_*a* values. **B** Lipid content (%) at each *k*_*L*_*a* value

To evaluate the effects of the *k*_*L*_*a* on biomass and lipid production in bioreactor cultivations, the *k*_*L*_*a* of 28.41 h^−1^, was established as the initial reference point. This value corresponded to an agitation speed of 207 rpm in the bioreactor. The agitation was determined using an equation derived from *k*_*L*_*a* measurements performed via the gassing-out method in the selected bioreactor. The cultivations were performed in duplicate, and there was no statistical difference between treatments in terms of specific growth rate and final biomass production. The highest values of lipid content and lipid titer were recorded in *k*_*L*_*a* values of 49.42, and 103.15 h^−1^ (Fig. 6). Since there is no statistical difference between *k*_*L*_*a* values of 49.42 and 103.15 h^−1^ with regard to these lipid production parameters, the *k*_*L*_*a* of 49.42 h⁻^1^ was selected for lipid production by *P. laurentii*, as this condition requires lower agitation compared to 103.15 h⁻^1^. This represented a 691% increase in biomass production and an 815% increase in lipid titer and volumetric productivity compared to the reference condition.

Consistent with our results, the *k*_*L*_*a* is considered a critical parameter influencing lipid accumulation and lipid titers in microorganisms, as it directly affects the dissolved oxygen (DO) levels in the culture medium. Dissolved oxygen is known to significantly impact not only microbial growth but also lipid content and lipid profiles, playing a key role in regulating the physiological and metabolic characteristics in oleaginous yeasts [61, 62]. As a critical component of aerobic metabolism, oxygen is indispensable for sustaining the tricarboxylic acid (TCA) cycle, which generates NADH and FADH₂ essentially reducing equivalents for oxidative phosphorylation via the electron transport chain. Furthermore, acetyl-CoA, the building block for de novo fatty acid biosynthesis, accumulates proportionally to TCA cycle flux, a process directly regulated by oxygen availability [11, 63]. However, the effect of DO on lipid accumulation is species-dependent and varies among microorganisms. For instance, studies have shown that higher dissolved oxygen levels can lead to reduced lipid accumulation in *Yarrowia lipolytica* and *Rhodotorula glutinis* [64, 65]. In contrast, increased DO levels have been associated with enhanced lipid accumulation in *Apiotrichum curvatum*, *Scheffersomyces segobiensis,* and *Pichia guilliermondii* (*Meyerozyma guilliermondii*) [62, 66, 67], which is consistent with our results. In general, increased dissolved oxygen levels tend to promote higher lipid titers in oleaginous yeasts, either by enhancing cellular biomass, thereby increasing the number of lipid-accumulating cells in a given culture, or by elevating the lipid content itself. This observation is consistent with the findings reported in this study [62, 65, 66].

The underlying mechanisms for these differences are linked to the metabolic responses of yeasts to oxygen availability. In some yeasts, lower dissolved oxygen levels promote lipid accumulation by triggering a stress or starvation response, which activates adenosine monophosphate (AMP) deaminase [64]. This enzyme reduces AMP concentrations, leading to an increase in citrate levels. Citrate is a precursor for acetyl-CoA, a crucial substrate for fatty acid biosynthesis. In *Yarrowia lipolytica*, low aeration levels reduce the activity of mitochondrial enzymes involved in citric acid synthesis due to decreased electron transport chain flux, resulting in lower citrate production [11]. For yeasts in which reduced dissolved oxygen levels result in lower lipid content, a potential mechanism may involve the diminished production of citrate, whose accumulation is crucial for lipid biosynthesis. Since citrate accumulation is essential for lipogenesis, higher *k*_*L*_*a* values may enhance mitochondrial enzyme activity, increase citrate production, and consequently promote greater lipid accumulation. This could explain why certain yeasts exhibit higher lipid accumulation under elevated dissolved oxygen conditions. Moreover, oxygen is a key factor in energy metabolism and the synthesis of cellular components. A higher oxygen supply in the culture medium can improve energy metabolism and the synthesis of biomolecules, including lipids [22]. Therefore, optimizing *k*_*L*_*a* to achieve the appropriate dissolved oxygen levels is crucial for maximizing lipid accumulation and lipid titers in oleaginous yeasts, with the specific requirements varying depending on the microbial species.

Compared to other oleaginous yeasts, the volumetric lipid productivity of *P. laurentii* UFV-1 under optimized conditions surpassed other yeasts previously recognized as efficient lipid producers. Specifically, *Lipomyces starkeyi* NBRC 10381 and *Rhodotorula toruloides* NBRC 0559 achieved volumetric productivities of 0.14 g/L day and 0.12 g/L day, respectively, when cultivated in SS2 medium. In contrast, under optimized conditions in the SS2 medium modified in this work, *P. laurentii* UFV-1 presented a volumetric productivity of 2.27 g/L day. This represents a 16-fold increase over *L. starkeyi* NBRC 10381 and a 19-fold increase over *R. toruloides* NBRC 0559 which were cultivated in conventional SS2 medium [27].

## Conclusions

This was the first study to combine genome-scale metabolic modeling with factorial design and bioreactor optimization in *Papiliotrema laurentii* to improve both biomass and lipid production. Initially, flux balance analysis (FBA) was employed as a predictive tool to rationally select nutrient combinations that favored biomass production. Subsequently, CCRD was used to optimize cultivation parameters. Therefore, FBA combined with experimental optimization allows us to identify the key factors that maximize biomass and lipid production by P*. laurentii*. The best carbon and nitrogen source combination for enhancing both biomass and lipid titers was lactose and urea, respectively. Yeast extract at 0.5 g/L increases these titers. The *k*_*L*_*a* was the primary limiting factor for both biomass and lipid titer. Bioreactor cultivation conducted at *k*_*L*_*a* = 49.42 h⁻^1^ improved lipid production, resulting in increases of 691% and 815% in lipid titer and volumetric productivity, respectively. Therefore, *k*_*L*_*a* is a critical parameter for biomass and lipid production. Herein, FBA guided the definition of the culture medium composition for the *P. laurentii* cultivation. Future studies might exploit FBA to guide the Design-Build-Test-Learn (DBTL) cycle in the context of systems metabolic engineering, aiming at enhancing lipid production by *P. laurentii*.

## Supplementary Information

Below is the link to the electronic supplementary material.

Supplementary file1 (DOCX 129 KB)

Supplementary file2 (PDF 6 KB)

## Data Availability

Data will be made available on request.
