# Supplementary material for: Improvement of the production of biomass and lipid by Papiliotrema laurentii combining flux balance analysis and central composite rotational design
Source: Bioprocess Biosyst Eng. 2026 Mar 23;49(5):1233–48. doi: 10.1007/s00449-026-03311-z (PMC13263264; doi:10.1007/s00449-026-03311-z)
Supplement: Supplementary file 1 — Supplementary file1 (DOCX 129 KB) [file 449_2026_3311_MOESM1_ESM.docx]

SUPPLEMENTARY FILE

**Improvement of the Production of Biomass and Lipid by *Papiliotrema laurentii* Combining Flux Balance Analysis and Central Compound Rotational Design**

**Samuel Lessa Barbosa^a^, Eduardo Luís Menezes de Almeida^a^, Rafaela Zandonade Ventorim^a^, Jimmy Soares^b^, Wendel Batista da Silveira^a*^**

*^a^Laboratory of Microbial Physiology, Department of Microbiology, Universidade Federal de Viçosa, Viçosa, Brazil
^b^Secretaria de Estado de Educação de Minas Gerais, Minas Gerais, Brazil*

*Corresponding author: Laboratory of Microbial Physiology, Department of Microbiology, Universidade Federal de Viçosa, Viçosa, Brasil. E-mail address: [wendel.silveira@ufv.br](mailto:wendel.silveira@ufv.br); [samuel.lessa@ufv.br](mailto:samuel.lessa@ufv.br); [eduardo.menezes@ufv.br](mailto:eduardo.menezes@ufv.br); [rafaela.ventorim@ufv.br](mailto:rafaela.ventorim@ufv.br); jimmy_soares@aol.com.

SUPPLEMENTARY FIGURE


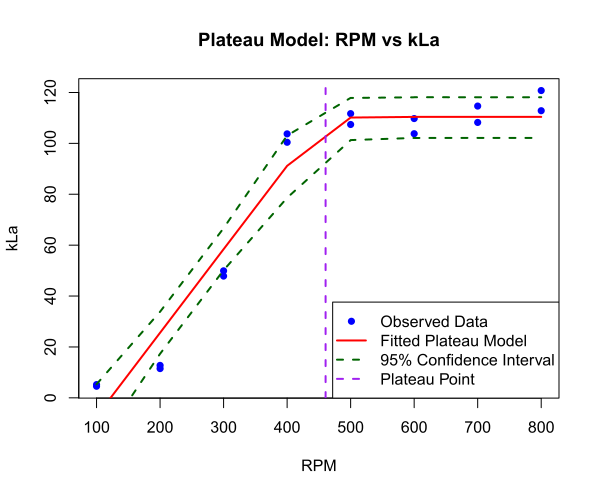


**Supplementary Figure 1**: Relationship between the volumetric mass transfer coefficient (*k_La_*) and agitation rate (RPM) at a fixed aeration rate of 0.5 vvm. Adjusted plateau response model of kLa as a function of agitation. Plateau model equation: kLa = -40.09 + 0.33 × RPM (if RPM ≤ 460.58); kLa = 111.14 (if RPM > 460.58). R² of the plateau model: 0.9591.

SUPPLEMENTARY TABLE

**Supplementary Table 1** – List of modified reactions in *papla-*GEM.

| **ID** | **Name** | **Equation** | **EC** | **Gene Association** |
| --- | --- | --- | --- | --- |
| r_0001 | (R)-lactate:ferricytochrome-c 2-oxidoreductase | (R)-lactate[c] + 2 ferricytochrome c[m] => 2 ferrocytochrome c[m] + pyruvate[c] | 1.1.2.4;1.1.99.- | PAPLA_00446 or PAPLA_01906 or PAPLA_02186 or PAPLA_03705 |
| r_0002 | (R)-lactate:ferricytochrome-c 2-oxidoreductase | (R)-lactate[m] + 2 ferricytochrome c[m] => 2 ferrocytochrome c[m] + pyruvate[m] | 1.1.2.4;1.1.99.- | (PAPLA_03705 and PAPLA_05450) or (PAPLA_02186 and PAPLA_05450) |
| r_0004 | (S)-lactate:ferricytochrome-c 2-oxidoreductase | (S)-lactate[c] + 2 ferricytochrome c[m] => 2 ferrocytochrome c[m] + pyruvate[c] | 1.1.2.3 | (PAPLA_05450 and PAPLA_01825) or (PAPLA_05450 and PAPLA_03690) or (PAPLA_05450 and PAPLA_04162) or (PAPLA_05450 and PAPLA_04267) or (PAPLA_05450 and PAPLA_04342) or (PAPLA_05450 and PAPLA_04700) or (PAPLA_05450 and PAPLA_04894) |
| r_0013 | 2,3-diketo-5-methylthio-1-phosphopentane degradation reaction | 5-(methylsulfanyl)-2,3-dioxopentyl phosphate[c] + 3 H2O[c] => 4-methylthio-2-oxobutanoate[c] + formate[c] + 6 H+[c] + phosphate[c] | 1.13.11.54;3.1.3.77 | PAPLA_01699 |
| r_0014 | 2,5-diamino-6-ribitylamino-4(3H)-pyrimidinone 5'-phosphate deaminase | 2,5-diamino-6-(5-phosphono)ribitylamino-4(3H)-pyrimidinone[c] + H+[c] + H2O[c] => 5-amino-6-(5-phosphoribitylamino)uracil[c] + ammonium[c] + 2 H+[c] | 3.5.4.26;5.4.99.28 | PAPLA_02908 |
| r_0015 | 2,5-diamino-6-ribosylamino-4(3H)-pyrimidinone 5'-phosphate reductase (NADPH) | 2,5-diamino-4-hydroxy-6-(5-phosphoribosylamino)pyrimidine[c] + 3 H+[c] + NADPH[c] => 2,5-diamino-6-(5-phosphono)ribitylamino-4(3H)-pyrimidinone[c] + NADP(+)[c] | 1.1.1.302 | PAPLA_05408 |
| r_0017 | 2-amino-4-hydroxy-6-hydroxymethyldihydropteridine diphosphokinase | 2-amino-6-(hydroxymethyl)-7,8-dihydropteridin-4-ol[m] + ATP[m] => (2-amino-4-hydroxy-7,8-dihydropteridin-6-yl)methyl trihydrogen diphosphate[m] + AMP[m] + H+[m] | 2.5.1.15;2.7.6.3;4.1.2.25 | PAPLA_05610 |
| r_0034 | 3',5'-cyclic-nucleotide phosphodiesterase | 3',5'-cyclic dAMP[c] + H2O[c] => dAMP[c] + 2 H+[c] | 3.1.4.53 | PAPLA_03615 |
| r_0035 | 3',5'-cyclic-nucleotide phosphodiesterase | 3',5'-cyclic IMP[c] + H2O[c] => 2 H+[c] + IMP[c] | 3.1.4.53 | PAPLA_03615 |
| r_0038 | 3,4-dihydroxy-2-butanone-4-phosphate synthase | D-ribulose 5-phosphate[c] => 2-hydroxy-3-oxobutyl phosphate[c] + formate[c] + 3 H+[c] | 4.1.99.12 | PAPLA_05155 |
| r_0041 | 3-dehydrosphinganine reductase | 3-ketosphinganine[er] + H+[er] + NADPH[er] => NADP(+)[er] + sphinganine[er] | 1.1.1.102 | PAPLA_02031 |
| r_0043 | 3-hexaprenyl-4,5-dihydroxybenzoate hydroxylase | 3-hexaprenyl-4-hydroxybenzoic acid[c] + 0.5 oxygen[c] => 3-hexaprenyl-4,5-dihydroxybenzoic acid[c] + H+[c] |  | PAPLA_04807 |
| r_0045 | 3-hydroxy-L-kynurenine hydrolase | 3-hydroxy-L-kynurenine[c] + H2O[c] => 3-hydroxyanthranilate[c] + L-alanine[c] + H+[c] | 3.7.1.3 | PAPLA_02789 |
| r_0063 | 3-methyl-2-oxobutanoate hydroxymethyltransferase | 3-methyl-2-oxobutanoate[m] + 5,10-methylenetetrahydrofolate[m] + H2O[m] => 2-dehydropantoate[m] + THF[m] + H+[m] | 2.1.2.11 | PAPLA_01102 |
| r_0066 | 4-amino-4-deoxychorismate synthase | chorismate[c] + L-glutamine[c] => 4-amino-4-deoxychorismate[c] + L-glutamate[c] + H[c] | 2.6.1.85 | PAPLA_01449 |
| r_0067 | 4-aminobenzoate synthase | 4-amino-4-deoxychorismate[c] + H+[c] => 4-aminobenzoate[c] + H+[c] + pyruvate[c] | 4.1.3.38 | PAPLA_03832 |
| r_0073 | 4PP-IP5 depyrophosphorylation to IP6 | 4-diphospho-1D-myo-inositol pentakisphosphate[c] + H2O[c] <=> 2 H+[c] + myo-inositol hexakisphosphate[c] + phosphate[c] + 12H+[c] | 2.7.4.21;2.7.4.24;3.6.1.52;3.6.1.60 | PAPLA_00179 |
| r_0080 | 5,10-methylenetetrahydrofolate reductase (NADPH) | 5,10-methylenetetrahydrofolate[c] + H+[c] + NADPH[c] => 5-methyltetrahydrofolate[c] + NADP(+)[c] | 1.5.1.20 | PAPLA_04325 or PAPLA_05344 |
| r_0088 | 5PP-IP5 pyrophosphorylation to 4,5-PP2-IP4 | 5-diphospho-1D-myo-inositol pentakisphosphate[c] + 15 H+[c] + phosphate[c] <=> 4,5-bis(diphospho)-1D-myo-inositol tetrakisphosphate[c] + H2O[c] | 2.7.4.21;2.7.4.24 | PAPLA_00179 |
| r_0089 | 5PP-IP5 pyrophosphorylation to 5,6-PP2-IP4 | 5-diphospho-1D-myo-inositol pentakisphosphate[c] + 15 H+[c] + phosphate[c] <=> 5,6-bis(diphospho)-1D-myo-inositol tetrakisphosphate[c] + H2O[c] | 2.7.4.21;2.7.4.24 | PAPLA_00179 |
| r_0092 | 6PP-IP5 depyrophosphorylation to IP6 | 6-diphospho-1D-myo-inositol pentakisphosphate[c] + H2O[c] <=> 14 H+[c] + myo-inositol hexakisphosphate[c] + phosphate[c] | 2.7.4.21;2.7.4.24;3.6.1.52;3.6.1.60 | PAPLA_00179 |
| r_0099 | acetyl-CoA ACP transacylase | acetyl-CoA[m] + ACP1[m] <=> acetyl-ACP[m] + coenzyme A[m] |  | PAPLA_03341 |
| r_0100 | acetyl-CoA C-acyltransferase (palmitoyl-CoA) | 3-oxooctadecanoyl-CoA[p] + coenzyme A[p] + 2 H+[p] => acetyl-CoA[p] + palmitoyl-CoA[p] | 2.3.1.16 | PAPLA_00899 or PAPLA_01492 or PAPLA_01644 |
| r_0105 | acetyl-CoA C-acyltransferase (lauroyl-CoA) | 3-oxotetradecanoyl-CoA[p] + coenzyme A[p] => acetyl-CoA[p] + lauroyl-CoA[p] + 4 H+[p] | 2.3.1.16 | PAPLA_00899 or PAPLA_01492 or PAPLA_01644 |
| r_0106 | acetyl-CoA C-acyltransferase (octanoyl-CoA) | 3-oxodecanoyl-CoA[p] + coenzyme A[p] => acetyl-CoA[p] + octanoyl-CoA[p] + 4 H+[p] | 2.3.1.16 | PAPLA_00899 or PAPLA_01492 or PAPLA_01644 |
| r_0107 | acetyl-CoA C-acyltransferase (decanoyl-CoA) | 3-oxolauroyl-CoA[p] + coenzyme A[p] => acetyl-CoA[p] + decanoyl-CoA[p] + 4 H+[p] | 2.3.1.16 | PAPLA_00899 or PAPLA_01492 or PAPLA_01644 |
| r_0123 | acyl-CoA oxidase (hexadecanoyl-CoA) | oxygen[p] + palmitoyl-CoA[p] + 4 H+[p] => hexadec-2-enoyl-CoA[p] + hydrogen peroxide[p] | 1.3.3.6 | PAPLA_02012 or PAPLA_02013 |
| r_0124 | acyl-CoA oxidase (octadecanoyl-CoA) | oxygen[p] + stearoyl-CoA[p] + 4 H+[p] => hydrogen peroxide[p] + trans-octadec-2-enoyl-CoA[p] | 1.3.3.6 | PAPLA_02012 or PAPLA_02013 |
| r_0137 | acylation of GPI inositol at 2 position, GPI-anchor assembly, step 3 | 6-(alpha-D-glucosaminyl)-1-phosphatidyl-1D-myo-inositol[er] + palmitoyl-CoA[er] <=> 6-(alpha-D-glucosaminyl)-O-acyl-1-phosphatidyl-1D-myo-inositol[er] + coenzyme A[er] | 2.3.-.- | PAPLA_06142 |
| r_0146 | adenosylmethionine-8-amino-7-oxononanoate transaminase | 8-amino-7-oxononanoate[c] + S-adenosyl-L-methionine[c] <=> 7,8-diaminononanoate[c] + S-adenosyl-4-methylthio-2-oxobutanoate[c] + 2 H+[c] | 2.6.1.62 | PAPLA_04295 |
| r_0150 | adenylate kinase (GTP) | AMP[m] + GTP[m] <=> ADP[m] + GDP[m] + H+[m] | 2.7.4.10 | PAPLA_02396 or PAPLA_02489 |
| r_0153 | adenylosuccinate synthase | GTP[c] + IMP[c] + L-aspartate[c] => adenylo-succinate[c] + GDP[c] + 3 H+[c] + phosphate[c] | 6.3.4.4 | PAPLA_00362 |
| r_0157 | alanyl-tRNA synthetase | ATP[c] + L-alanine[c] + tRNA(Ala)[c] => Ala-tRNA(Ala)[c] + AMP[c] + diphosphate[c] | 6.1.1.7 | PAPLA_05796 |
| r_0189 | allantoate amidinohydrolase | allantoate[c] + H2O[c] + H+[c] <=> urea[c] + ureidoglycolic acid[c] | 3.5.3.4 | PAPLA_02905 |
| r_0195 | alpha,alpha-trehalose-phosphate synthase (UDP-forming) | D-glucose 6-phosphate[c] + UDP-D-glucose[c] => alpha,alpha-trehalose 6-phosphate[c] + 3 H+[c] + UDP[c] | 2.4.1.15;3.1.3.12 | PAPLA_01725 or PAPLA_03918 |
| r_0209 | arginyl-tRNA synthetase | ATP[c] + L-arginine[c] + tRNA(Arg)[c] => AMP[c] + Arg-tRNA(Arg)[c] + diphosphate[c] | 6.1.1.19 | PAPLA_00408 |
| r_0210 | arginyl-tRNA synthetase | ATP[m] + L-arginine[m] + tRNA(Arg)[m] => AMP[m] + Arg-tRNA(Arg)[m] + diphosphate[m] | 6.1.1.19 | PAPLA_00408 |
| r_0212 | Asparaginyl-tRNA synthetase | ATP[c] + L-asparagine[c] + tRNA(Asn)[c] => AMP[c] + Asn-tRNA(Asn)[c] + diphosphate[c] | 6.1.1.22 | PAPLA_05069 |
| r_0213 | asparaginyl-tRNA synthetase, miotchondrial | ATP[m] + L-asparagine[m] + tRNA(Asn)[m] => AMP[m] + Asn-tRNA(Asn)[m] + diphosphate[m] | 6.1.1.22 | PAPLA_03077 |
| r_0215 | aspartate kinase | ATP[c] + L-aspartate[c] => 4-phospho-L-aspartate[c] + ADP[c] + H+[c] | 2.7.2.4 |  |
| r_0219 | aspartate-semialdehyde dehydrogenase | 4-phospho-L-aspartate[c] + 2 H+[c] + NADPH[c] => L-aspartate 4-semialdehyde[c] + NADP(+)[c] + phosphate[c] | 1.2.1.11 | PAPLA_04388 |
| r_0228 | beta-1,4 mannosyltransferase | GDP-alpha-D-mannose[c] + N,N'-diacetylchitobiosyldiphosphodolichol[c] => beta-D-mannosyldiacetylchitobiosyldiphosphodolichol[c] + GDP[c] + H+[c] | 2.4.1.142 | PAPLA_00090 |
| r_0229 | biotin synthase | dethiobiotin[c] + polysulphur[c] <=> biotin[c] + 2 H+[c] | 2.8.1.6 | PAPLA_05690 |
| r_0234 | C-3 sterol dehydrogenase | NADP(+)[c] + zymosterol intermediate 1c[c] => carbon dioxide[c] + NADPH[c] + zymosterol intermediate 2[c] | 1.1.1.170 | PAPLA_01122 |
| r_0240 | C-4 methyl sterol oxidase | NADPH[c] + oxygen[c] + zymosterol intermediate 1b[c] => H2O[c] + NADP(+)[c] + zymosterol intermediate 1c[c] | 1.14.13.72 | PAPLA_03797 |
| r_0263 | ceramide-1 synthase (24C) | sphinganine[er] + tetracosanoyl-CoA[er] => ceramide-1 (C24)[er] + coenzyme A[er] + 5 H+[er] | 2.3.1.24 | PAPLA_00132 |
| r_0264 | ceramide-1 synthase (26C) | hexacosanoyl-CoA[er] + sphinganine[er] => ceramide-1 (C26)[er] + coenzyme A[er] + 4 H+[er] | 2.3.1.24 | PAPLA_00132 |
| r_0265 | ceramide-2 synthase (24C) | phytosphingosine[er] + tetracosanoyl-CoA[er] => ceramide-2 (C24)[er] + coenzyme A[er] + 4 H+[er] | 2.3.1.24 | PAPLA_00132 |
| r_0266 | ceramide-2 synthase (26C) | hexacosanoyl-CoA[er] + phytosphingosine[er] => ceramide-2 (C26)[er] + coenzyme A[er] + 4 H+[er] | 2.3.1.24 | PAPLA_00132 |
| r_0271 | chitin deacetylase | chitin[c] + H2O[c] => acetate[c] + chitosan[ce] + H+[c] | 3.5.1.41 | PAPLA_03127 or PAPLA_05978 |
| r_0274 | choline phosphate cytididyltransferase | choline phosphate[c] + CTP[c] + 2 H+[c] => CDP-choline[c] + diphosphate[c] | 2.7.7.15 | PAPLA_01626 |
| r_0282 | cis-prenyltransferase step 02 | geranylgeranyl diphosphate[lp] + isopentenyl diphosphate[lp] + 3 H+ => diphosphate[lp] + pentaprenyl diphosphate[lp] | 2.5.1.87 | PAPLA_03087 |
| r_0312 | cysteine synthase | hydrogen sulfide[c] + O-acetyl-L-serine[c] + H+[c] => acetate[c] + H+[c] + L-cysteine[c] | 2.5.1.47;2.5.1.49 | PAPLA_05226 |
| r_0313 | cysteinyl-tRNA synthetase | ATP[c] + L-cysteine[c] + tRNA(Cys)[c] => AMP[c] + Cys-tRNA(Cys)[c] + diphosphate[c] | 6.1.1.16 | PAPLA_03667 |
| r_0315 | cytidine kinase (GTP) | cytidine[c] + GTP[c] => CMP[c] + GDP[c] + 2 H+[c] | 2.7.1.48 | PAPLA_00418 |
| r_0322 | D-fructose 1-phosphate D-glyceraldehyde-3-phosphate-lyase | D-fructose 1-phosphate[c] <=> D-glyceraldehyde[c] + dihydroxyacetone phosphate[c] + 2 H+[c] | 4.1.2.13 | PAPLA_03603 |
| r_0344 | dihydrofolate reductase | dihydrofolic acid[c] + 3 H+[c] + NADPH[c] => NADP(+)[c] + THF[c] | 1.5.1.3 |  |
| r_0345 | dihydrofolate reductase | dihydrofolic acid[m] + 3 H+[m] + NADPH[m] => NADP(+)[m] + THF[m] | 1.5.1.3 |  |
| r_0350 | dihydropteroate synthase | 2-amino-6-(hydroxymethyl)-7,8-dihydropteridin-4-ol[m] + 4-aminobenzoate[m] => 7,8-dihydropteroate[m] + H2O[m] | 2.5.1.15;2.7.6.3;4.1.2.25 | PAPLA_05610 |
| r_0351 | dihydropteroate synthase | (2-amino-4-hydroxy-7,8-dihydropteridin-6-yl)methyl trihydrogen diphosphate[m] + 4-aminobenzoate[m] => 7,8-dihydropteroate[m] + diphosphate[m] | 2.5.1.15;2.7.6.3;4.1.2.25 | PAPLA_05610 |
| r_0356 | diphosphoglyceromutase | 1,3-bisphospho-D-glycerate[c] <=> 2,3-bisphospho-D-glyceric acid[c] + H+[c] | 5.4.2.11 |  |
| r_0357 | diphosphoinositol-1,3,4,6-tetrakisphosphate diphosphohydrolase | 5,6-bis(diphospho)-1D-myo-inositol tetrakisphosphate[c] + 3 H2O[c] => 16 H+[c] + myo-inositol 1,3,4,5,6-pentakisphosphate[c] + 3 phosphate[c] | 3.6.1.52;3.6.1.60 |  |
| r_0360 | dolichol kinase | CTP[c] + dolichol[c] + 2 H+[c] => CDP[c] + dolichyl phosphate[c] + H+[c] | 2.7.1.108 | PAPLA_04079 |
| r_0361 | dolichyl-phosphate D-mannosyltransferase | dolichyl phosphate[c] + GDP-alpha-D-mannose[c] => dolichyl D-mannosyl phosphate[er] + GDP[c] + H+[c] | 2.4.1.83 | PAPLA_03913 |
| r_0362 | dolichyl-phosphate-mannose--protein mannosyltransferase | dolichyl D-mannosyl phosphate[er] => dolichyl phosphate[er] + H+[er] + mannan[er] | 2.4.1.109 | PAPLA_02436 or PAPLA_02894 |
| r_0363 | dTMP kinase | ATP[c] + dTMP[c] => ADP[c] + dTDP[c] + 2 H+[c] | 2.7.4.9 | PAPLA_04551 |
| r_0364 | dUTP diphosphatase | dUTP[c] + H2O[c] => diphosphate[c] + dUMP[c] + 2 H+[c] | 3.6.1.23 | PAPLA_04480 |
| r_0366 | enolase | 2-phospho-D-glyceric acid[c] <=> H2O[c] + phosphoenolpyruvate[c] | 4.2.1.11 |  |
| r_0368 | ethanolamine kinase | ATP[c] + ethanolamine[c] => ADP[c] + H+[c] + O-phosphoethanolamine[c] | 2.7.1.32;2.7.1.82 | PAPLA_06289 |
| r_0438 | ferrocytochrome-c:oxygen oxidoreductase | ferrocytochrome c[m] + 1.266 H+[m] + 0.25 oxygen[m] => ferricytochrome c[m] + 0.633 H+[c] + 0.5 H2O[m] | 1.9.3.1 | COX1 and COX2 and COX3 and PAPLA_05450 and PAPLA_04298 and PAPLA_00749 and PAPLA_03809 and PAPLA_05941 |
| r_0439 | ubiquinol:ferricytochrome c reductase | 2 ferricytochrome c[m] + 1.266 H+[m] + ubiquinol-6[m] => 2 ferrocytochrome c[m] + 2.532 H+[c] + ubiquinone-6[m] | 1.10.2.2 | PAPLA_00367 and PAPLA_05898 and PAPLA_04311 |
| r_0446 | formate-tetrahydrofolate ligase | ATP[c] + formate[c] + THF[c] <=> 10-formyl-THF[c] + ADP[c] + phosphate[c] + 2 H+[c] | 1.5.1.5;3.5.4.9;6.3.4.3 | PAPLA_00443 |
| r_0447 | formate-tetrahydrofolate ligase | ATP[m] + formate[m] + THF[m] <=> 10-formyl-THF[m] + ADP[m] + phosphate[m] + 2 H+[m] | 1.5.1.5;3.5.4.9;6.3.4.3 | PAPLA_00443 |
| r_0449 | fructose-bisphosphatase | D-fructose 1,6-bisphosphate[c] + H2O[c] => D-fructose 6-phosphate[c] + phosphate[c] + 4 H+[c] | 3.1.3.11 | PAPLA_04632 |
| r_0450 | fructose-bisphosphate aldolase | D-fructose 1,6-bisphosphate[c] <=> dihydroxyacetone phosphate[c] + glyceraldehyde 3-phosphate[c] + 4 H+[c] | 4.1.2.13 | PAPLA_03603 |
| r_0454 | fumarate reductase | FADH2[m] + fumarate[m] <=> FAD[m] + succinate[m] + H+[m] | 1.3.1.6 | PAPLA_01100 |
| r_0455 | soluble fumarate reductase | FADH2[c] + fumarate[c] <=> FAD[c] + succinate[c] + H+[c] | 1.3.1.6 | PAPLA_01100 |
| r_0457 | g-glutamyltransferase | glutathione[c] + L-alanine[c] + H+[c] => L-cysteinylglycine[c] + L-gamma-glutamyl-L-alanine[c] | 2.3.2.2;3.4.19.13 | PAPLA_02433 |
| r_0463 | glucan 1,4-alpha-glucosidase | glycogen[c] + H2O[c] => D-glucose[c] | 2.4.1.25;3.2.1.33 | PAPLA_01354 |
| r_0467 | glucose-6-phosphate isomerase | D-glucose 6-phosphate[c] <=> D-fructose 6-phosphate[c] + 2 H+[c] | 5.3.1.9 | PAPLA_03864 |
| r_0477 | glutamine-fructose-6-phosphate transaminase | D-fructose 6-phosphate[c] + L-glutamine[c] + H+[c] => alpha-D-glucosamine 6-phosphate[c] + L-glutamate[c] | 2.6.1.16 | PAPLA_03131 |
| r_0478 | glutaminyl-tRNA synthetase | ATP[c] + L-glutamine[c] + tRNA(Gln)[c] => AMP[c] + diphosphate[c] + Gln-tRNA(Gln)[c] | 6.1.1.18 | PAPLA_05865 |
| r_0490 | glycerol-3-phosphate dehydrogenase (fad) | FAD[m] + glycerol 3-phosphate[m] + H+[m] => dihydroxyacetone phosphate[m] + FADH2[m] | 1.1.5.3 | PAPLA_01023 |
| r_0499 | glycinamide ribotide transformylase | 10-formyl-THF[c] + 5-phospho-ribosyl-glycineamide[c] => 5'-phosphoribosyl-N-formylglycineamide[c] + H+[c] + THF[c] + H+[c] | 2.1.2.2 |  |
| r_0501 | glycine cleavage system | L-glycine[m] + NAD[m] + THF[m] => 5,10-methylenetetrahydrofolate[m] + ammonium[m] + carbon dioxide[m] + NADH[m] + 2 H+[m] | 1.4.4.2;1.8.1.4;2.1.2.10 | PAPLA_06127 and PAPLA_02484 and PAPLA_00539 |
| r_0502 | glycine hydroxymethyltransferase | L-serine[c] + THF[c] <=> 5,10-methylenetetrahydrofolate[c] + H2O[c] + L-glycine[c] + 2 H+[c] | 2.1.2.1 | PAPLA_01106 |
| r_0503 | glycine hydroxymethyltransferase | L-serine[m] + THF[m] <=> 5,10-methylenetetrahydrofolate[m] + H2O[m] + L-glycine[m] 2 H+[m] | 2.1.2.1 | PAPLA_01106 |
| r_0504 | glycine-cleavage complex (lipoamide) | H+[m] + L-glycine[m] + lipoamide[m] => carbon dioxide[m] + S(8)-aminomethyldihydrolipoamide[m] + H+[m] | 1.4.4.2;1.8.1.4;2.1.2.10 | PAPLA_06127 and PAPLA_02484 and PAPLA_00539 |
| r_0506 | glycine-cleavage complex (lipoylprotein) | L-glycine[m] + lipoylprotein[m] => carbon dioxide[m] + S(8)-aminomethyldihydrolipoylprotein[m] | 1.4.4.2;1.8.1.4;2.1.2.10 | PAPLA_06127 and PAPLA_02484 and PAPLA_00539 |
| r_0507 | glycine-cleavage complex (lipoylprotein) | S(8)-aminomethyldihydrolipoylprotein[m] + THF[m] => 5,10-methylenetetrahydrofolate[m] + ammonium[m] + dihydrolipoylprotein[m] + H+[m] | 1.4.4.2;1.8.1.4;2.1.2.10 | PAPLA_06127 and PAPLA_02484 and PAPLA_00539 |
| r_0508 | glycine-cleavage complex (lipoylprotein) | dihydrolipoylprotein[m] + NAD[m] => H+[m] + lipoylprotein[m] + NADH[m] | 1.4.4.2;1.8.1.4;2.1.2.10 | PAPLA_06127 and PAPLA_02484 and PAPLA_00539 |
| r_0509 | glycine-cleavage system (lipoamide) | S(8)-aminomethyldihydrolipoamide[m] + THF[m] => 5,10-methylenetetrahydrofolate[m] + ammonium[m] + dihydrolipoamide[m] + H+[m] | 1.4.4.2;1.8.1.4;2.1.2.10 | PAPLA_06127 and PAPLA_02484 and PAPLA_00539 |
| r_0510 | glycogen (starch) synthase | UDP-D-glucose[c] => glycogen[c] + H+[c] + UDP[c] | 2.4.1.11;2.4.1.186 | PAPLA_01726 |
| r_0511 | glycogen phosphorylase | glycogen[c] + phosphate[c] => D-glucose 1-phosphate[c] | 2.4.1.1 | PAPLA_05741 or PAPLA_05742 |
| r_0512 | glycyl-tRNA synthetase | ATP[c] + L-glycine[c] + tRNA(Gly)[c] => AMP[c] + diphosphate[c] + Gly-tRNA(Gly)[c] | 6.1.1.14 | PAPLA_05875 |
| r_0518 | GPI-anchor assembly, step 2 | 6-(N-acetyl-alpha-D-glucosaminyl)-1-phosphatidyl-1D-myo-inositol[er] + H2O[er] <=> 6-(alpha-D-glucosaminyl)-1-phosphatidyl-1D-myo-inositol[er] + acetate[er] + H+[er] | 3.5.1.89 | PAPLA_03035 |
| r_0524 | GTP cyclohydrolase I | GTP[c] + H2O[c] => 7,8-dihydroneopterin 3'-triphosphate[c] + formate[c] + 2 H+[c] | 3.5.4.16 | PAPLA_03876 |
| r_0525 | GTP cyclohydrolase II | GTP[c] + 3 H2O[c] => 2,5-diamino-4-hydroxy-6-(5-phosphoribosylamino)pyrimidine[c] + diphosphate[c] + formate[c] + 3 H+[c] | 3.5.4.25 | PAPLA_00497 |
| r_0530 | heme O monooxygenase | heme o[m] + NADH[m] + oxygen[m] => H2O[m] + heme a[m] + NAD[m] + H+[m] | 1.18.1.6 | PAPLA_04807 or PAPLA_05552 |
| r_0531 | Heme O synthase | farnesyl diphosphate[m] + ferroheme b[m] + H2O[m] + 2 H+[m] => diphosphate[m] + heme o[m] | 2.5.1.- | PAPLA_04291 |
| r_0532 | hexaprenyldihydroxybenzoate methyltransferase | 2-hexaprenyl-5-hydroxy-6-methoxy-3-methyl-1,4-benzoquinone[m] + H+[m] + S-adenosyl-L-methionine[m] => S-adenosyl-L-homocysteine[m] + ubiquinol-6[m] | 1.14.13.-;2.1.1.114;2.1.1.201;2.1.1.64;2.7.-.- | (PAPLA_02366 and PAPLA_05126 and PAPLA_06261 and PAPLA_02748 and PAPLA_04581 and PAPLA_04074) or (PAPLA_02366 and PAPLA_05126 and PAPLA_06261 and PAPLA_04409 and PAPLA_04581 and PAPLA_04074) |
| r_0534 | hexokinase (D-glucose:ATP) | ATP[c] + D-glucose[c] + 2 H+[c] => ADP[c] + D-glucose 6-phosphate[c] + H+[c] | 2.7.1.1;2.7.1.2 | PAPLA_01860 or PAPLA_03777 |
| r_0535 | hexokinase (D-mannose:ATP) | ATP[c] + D-mannose[c] + 2 H+[c] => ADP[c] + D-mannose 6-phosphate[c] + H+[c] | 2.7.1.1 | PAPLA_01860 or PAPLA_03777 |
| r_0539 | histidyl-tRNA synthetase | ATP[c] + L-histidine[c] + tRNA(His)[c] => AMP[c] + diphosphate[c] + His-tRNA(His)[c] | 6.1.1.21 | PAPLA_00615 |
| r_0540 | histidyl-tRNA synthetase | ATP[m] + L-histidine[m] + tRNA(His)[m] => AMP[m] + diphosphate[m] + His-tRNA(His)[m] | 6.1.1.21 | PAPLA_00615 |
| r_0545 | homoisocitrate dehydrogenase | homoisocitrate[m] + NAD[m] + H+[m] => 2-oxoadipic acid[m] + carbon dioxide[m] + H+[m] + NADH[m] | 1.1.1.87 | PAPLA_01506 or PAPLA_03179 |
| r_0550 | hydrogen peroxide reductase (thioredoxin) | hydrogen peroxide[c] + TRX1[c] => 2 H2O[c] + TRX1 disulphide[c] | 1.11.1.15 | PAPLA_00392 and PAPLA_04953 |
| r_0551 | hydrogen peroxide reductase (thioredoxin) | hydrogen peroxide[m] + TRX1[m] => 2 H2O[m] + TRX1 disulphide[m] | 1.11.1.15 | PAPLA_02203 |
| r_0555 | hydroxybenzoate octaprenyltransferase | 4-hydroxybenzoate[m] + hexaprenyl diphosphate[m] => 3-hexaprenyl-4-hydroxybenzoic acid[m] + diphosphate[m] + 2 H+[m] | 2.5.1.39 | PAPLA_00457 |
| r_0558 | hydroxymethylglutaryl CoA reductase | 3-hydroxy-3-methylglutaryl-CoA[c] + 2 H+[c] + 2 NADPH[c] => (R)-mevalonate[c] + coenzyme A[c] + 2 NADP(+)[c] + 5 H+[c] | 1.1.1.34 | PAPLA_02600 |
| r_0559 | hydroxymethylglutaryl CoA synthase | acetoacetyl-CoA[c] + acetyl-CoA[c] + H2O[c] + 5 H+[c] => 3-hydroxy-3-methylglutaryl-CoA[c] + coenzyme A[c] + H+[c] | 2.3.3.10 | PAPLA_04117 |
| r_0560 | hydroxymethylglutaryl CoA synthase | acetoacetyl-CoA[m] + acetyl-CoA[m] + H2O[m] + 5 H+[m] => 3-hydroxy-3-methylglutaryl-CoA[m] + coenzyme A[m] + H+[m] | 2.3.3.10 | PAPLA_04117 |
| r_0572 | inositol-1,3,4,5-triphosphate 6-kinase, nucleus | 1D-myo-inositol 1,3,4,5-tetrakisphosphate[n] + ATP[n] + H+[n] => ADP[n] + myo-inositol 1,3,4,5,6-pentakisphosphate[n] + 2 H+[n] | 2.7.1.151 | PAPLA_05651 |
| r_0573 | inositol-1,4,5,6- tetrakisphosphate 3-kinase, nucleus | 1D-myo-inositol 1,4,5,6-tetrakisphosphate[n] + ATP[n] + H+[n] => ADP[n] + myo-inositol 1,3,4,5,6-pentakisphosphate[n] + 2 H+[n] | 2.7.1.151 | PAPLA_05651 |
| r_0574 | inositol-1,4,5-triphosphate 6-kinase, nucleus | 1D-myo-inositol 1,4,5-trisphosphate[n] + ATP[n] + H+[n] => 1D-myo-inositol 1,4,5,6-tetrakisphosphate[n] + ADP[n] + 2 H+[n] | 2.7.1.151 | PAPLA_05651 |
| r_0575 | inositol-1,4,5-trisphosphate 3-kinase, nucleus | 1D-myo-inositol 1,4,5-trisphosphate[n] + ATP[n] + H+[n] => 1D-myo-inositol 1,3,4,5-tetrakisphosphate[n] + ADP[n] + 2 H+[n] | 2.7.1.151 | PAPLA_05651 |
| r_0616 | IPS phospholipase C | H2O[m] + inositol-P-ceramide A (C24)[m] => 1D-myo-inositol 3-phosphate[m] + ceramide-1 (C24)[m] + 2 H+[m] | 3.1.4.- | PAPLA_00489 |
| r_0617 | IPS phospholipase C | H2O[m] + inositol-P-ceramide A (C26)[m] => 1D-myo-inositol 3-phosphate[m] + ceramide-1 (C26)[m] + 2 H+[m] | 3.1.4.- | PAPLA_00489 |
| r_0618 | IPS phospholipase C | H2O[m] + inositol-P-ceramide B' (C24)[m] => 1D-myo-inositol 3-phosphate[m] + ceramide-2' (C24)[m] + 2 H+[m] | 3.1.4.- | PAPLA_00489 |
| r_0619 | IPS phospholipase C | H2O[m] + inositol-P-ceramide B' (C26)[m] => 1D-myo-inositol 3-phosphate[m] + ceramide-2' (C26)[m] + 2 H+[m] | 3.1.4.- | PAPLA_00489 |
| r_0620 | IPS phospholipase C | H2O[m] + inositol-P-ceramide B (C24)[m] => 1D-myo-inositol 3-phosphate[m] + ceramide-2 (C24)[m] + 2 H+[m] | 3.1.4.- | PAPLA_00489 |
| r_0621 | IPS phospholipase C | H2O[m] + inositol-P-ceramide B (C26)[m] => 1D-myo-inositol 3-phosphate[m] + ceramide-2 (C26)[m] + 2 H+[m] | 3.1.4.- | PAPLA_00489 |
| r_0622 | IPS phospholipase C | H2O[m] + inositol-P-ceramide C (C24)[m] => 1D-myo-inositol 3-phosphate[m] + ceramide-3 (C24)[m] + 2 H+[m] | 3.1.4.- | PAPLA_00489 |
| r_0623 | IPS phospholipase C | H2O[m] + inositol-P-ceramide C (C26)[m] => 1D-myo-inositol 3-phosphate[m] + ceramide-3 (C26)[m] + 2 H+[m] | 3.1.4.- | PAPLA_00489 |
| r_0624 | IPS phospholipase C | H2O[m] + inositol-P-ceramide D (C24)[m] => 1D-myo-inositol 3-phosphate[m] + ceramide-4 (C24)[m] + 2 H+[m] | 3.1.4.- | PAPLA_00489 |
| r_0625 | IPS phospholipase C | H2O[m] + inositol-P-ceramide D (C26)[m] => 1D-myo-inositol 3-phosphate[m] + ceramide-4 (C26)[m] + 2 H+[m] | 3.1.4.- | PAPLA_00489 |
| r_0646 | IPS phospholipase C | H2O[er] + inositol-P-ceramide A (C24)[er] => 1D-myo-inositol 1-phosphate[er] + ceramide-1 (C24)[er] + 2 H+[er] | 3.1.4.- | PAPLA_00489 |
| r_0647 | IPS phospholipase C | H2O[er] + inositol-P-ceramide A (C26)[er] => 1D-myo-inositol 1-phosphate[er] + ceramide-1 (C26)[er] + 2 H+[er] | 3.1.4.- | PAPLA_00489 |
| r_0648 | IPS phospholipase C | H2O[er] + inositol-P-ceramide B' (C24)[er] => 1D-myo-inositol 1-phosphate[er] + ceramide-2' (C24)[er] + 2 H+[er] | 3.1.4.- | PAPLA_00489 |
| r_0649 | IPS phospholipase C | H2O[er] + inositol-P-ceramide B' (C26)[er] => 1D-myo-inositol 1-phosphate[er] + ceramide-2' (C26)[er] + 2 H+[er] | 3.1.4.- | PAPLA_00489 |
| r_0650 | IPS phospholipase C | H2O[er] + inositol-P-ceramide B (C24)[er] => 1D-myo-inositol 1-phosphate[er] + ceramide-2 (C24)[er] + 2 H+[er] | 3.1.4.- | PAPLA_00489 |
| r_0651 | IPS phospholipase C | H2O[er] + inositol-P-ceramide B (C26)[er] => 1D-myo-inositol 1-phosphate[er] + ceramide-2 (C26)[er] + 2 H+[er] | 3.1.4.- | PAPLA_00489 |
| r_0652 | IPS phospholipase C | H2O[er] + inositol-P-ceramide C (C24)[er] => 1D-myo-inositol 1-phosphate[er] + ceramide-3 (C24)[er] + 2 H+[er] | 3.1.4.- | PAPLA_00489 |
| r_0653 | IPS phospholipase C | H2O[er] + inositol-P-ceramide C (C26)[er] => 1D-myo-inositol 1-phosphate[er] + ceramide-3 (C26)[er] + 2 H+[er] | 3.1.4.- | PAPLA_00489 |
| r_0654 | IPS phospholipase C | H2O[er] + inositol-P-ceramide D (C24)[er] => 1D-myo-inositol 1-phosphate[er] + ceramide-4 (C24)[er] + 2 H+[er] | 3.1.4.- | PAPLA_00489 |
| r_0655 | IPS phospholipase C | H2O[er] + inositol-P-ceramide D (C26)[er] => 1D-myo-inositol 1-phosphate[er] + ceramide-4 (C26)[er] + 2 H+[er] | 3.1.4.- | PAPLA_00489 |
| r_0665 | isoleucyl-tRNA synthetase | ATP[c] + L-isoleucine[c] + tRNA(Ile)[c] => AMP[c] + diphosphate[c] + Ile-tRNA(Ile)[c] | 6.1.1.5 | PAPLA_03404 |
| r_0666 | isoleucyl-tRNA synthetase | ATP[m] + L-isoleucine[m] + tRNA(Ile)[m] => AMP[m] + diphosphate[m] + Ile-tRNA(Ile)[m] | 6.1.1.5 | PAPLA_03404 |
| r_0681 | L-erythro-4-hydroxyglutamate:2-oxoglutarate aminotransferase | 2-oxoglutarate[c] + erythro-4-hydroxy-L-glutamic acid[c] => 4-hydroxy-2-oxoglutarate[c] + L-glutamate[c] + H+[c] | 2.6.1.1 | PAPLA_05474 or PAPLA_05606 |
| r_0682 | L-erythro-4-hydroxyglutamate:2-oxoglutarate aminotransferase | 2-oxoglutarate[m] + erythro-4-hydroxy-L-glutamic acid[m] => 4-hydroxy-2-oxoglutarate[m] + L-glutamate[m] + H+[c] | 2.6.1.1 | PAPLA_05055 or PAPLA_05474 or PAPLA_05606 |
| r_0683 | L-erythro-4-hydroxyglutamate:2-oxoglutarate aminotransferase | 2-oxoglutarate[p] + erythro-4-hydroxy-L-glutamic acid[p] => 4-hydroxy-2-oxoglutarate[p] + L-glutamate[p] + H+[c] | 2.6.1.1 | PAPLA_05474 or PAPLA_05606 |
| r_0687 | L-hydroxyproline reductase (NADP) | 1-pyrroline-3-hydroxy-5-carboxylic acid[c] + 2 H+[c] + NADPH[c] => L-proline[c] + NADP(+)[c] | 1.5.1.2 | PAPLA_05909 |
| r_0690 | L-serine dehydrogenase | L-serine[c] + NADP(+)[c] => 2 H+[c] + L-alpha-formylglycine[c] + NADPH[c] | 1.1.1.381 | PAPLA_03698 |
| r_0701 | leucyl-tRNA synthetase | ATP[c] + L-leucine[c] + tRNA(Leu)[c] => AMP[c] + diphosphate[c] + Leu-tRNA(Leu)[c] | 6.1.1.4 | PAPLA_03080 |
| r_0711 | lysyl-tRNA synthetase | ATP[c] + L-lysine[c] + tRNA(Lys)[c] => AMP[c] + diphosphate[c] + Lys-tRNA(Lys)[c] | 6.1.1.6 | PAPLA_06162 |
| r_0712 | lysyl-tRNA synthetase | ATP[m] + L-lysine[m] + tRNA(Lys)[m] => AMP[m] + diphosphate[m] + Lys-tRNA(Lys)[m] | 6.1.1.6 | PAPLA_06162 |
| r_0722 | mannose-1-phosphate guanylyltransferase | D-mannose 1-phosphate[c] + GTP[c] + H+[c] => diphosphate[c] + GDP-alpha-D-mannose[c] + 3 H+[c] | 2.7.7.13 |  |
| r_0723 | mannose-6-phosphate isomerase | D-mannose 6-phosphate[c] <=> D-fructose 6-phosphate[c] + 2 H+[c] | 5.3.1.8 | PAPLA_05632 |
| r_0727 | methionine synthase | 5-methyltetrahydrofolate[c] + L-homocysteine[c] + 3 H+[c]=> H+[c] + L-methionine[c] + THF[c] | 2.1.1.14 | PAPLA_02319 |
| r_0729 | methionyl-tRNA synthetase | ATP[c] + L-methionine[c] + tRNA(Met)[c] => AMP[c] + diphosphate[c] + Met-tRNA(Met)[c] | 6.1.1.10 | PAPLA_03425 |
| r_0730 | methionyl-tRNA synthetase | ATP[m] + L-methionine[m] + tRNA(Met)[m] => AMP[m] + diphosphate[m] + Met-tRNA(Met)[m] | 6.1.1.10 | PAPLA_04616 |
| r_0737 | mevalonate kinase (gtp) | (R)-mevalonate[c] + GTP[c] => (R)-5-phosphomevalonic acid[c] + GDP[c] + 2 H+[c] | 2.7.1.36 | PAPLA_02274 |
| r_0758 | myo-inositol-1-phosphate synthase | D-glucose 6-phosphate[c] => 1D-myo-inositol 1-phosphate[c] + 2 H+[c] | 5.5.1.4 | PAPLA_00616 |
| r_0760 | N-acetylglucosamine-6-phosphate synthase | acetyl-CoA[c] + alpha-D-glucosamine 6-phosphate[c] <=> coenzyme A[c] + 4 H+[c] + N-acetyl-D-glucosamine 6-phosphate[c] | 2.3.1.4 | PAPLA_05731 or PAPLA_05780 |
| r_0768 | NAD synthase | ATP[c] + deamido-NAD(+)[c] + H2O[c] + L-glutamine[c] => AMP[c] + diphosphate[c] + 4 H+[c] + L-glutamate[c] + NAD[c] | 6.3.5.1 |  |
| r_0769 | NAD synthase | ATP[n] + deamido-NAD(+)[n] + H2O[n] + L-glutamine[n] <=> AMP[n] + diphosphate[n] + 4 H+[n] + L-glutamate[n] + NAD[n] | 6.3.5.1 |  |
| r_0782 | nicotinamide N-methyltransferase | nicotinamide[c] + S-adenosyl-L-methionine[c] => 1-methylnicotinamide[c] + S-adenosyl-L-homocysteine[c] | 2.1.1.- | PAPLA_00307 |
| r_0785 | nicotinate-nucleotide adenylyltransferase | ATP[c] + 4 H+[c] + nicotinic acid D-ribonucleotide[c] => deamido-NAD(+)[c] + diphosphate[c] | 2.7.7.1;2.7.7.18 | PAPLA_05907 |
| r_0797 | nucleoside diphosphate kinase | ATP[c] + dCDP[c] + H+[c] => ADP[c] + dCTP[c] | 2.7.4.6 | PAPLA_01099 or PAPLA_04870 |
| r_0798 | nucleoside diphosphate kinase | ATP[c] + dGDP[c] + H+[c] => ADP[c] + dGTP[c] | 2.7.4.6 | PAPLA_01099 or PAPLA_04870 |
| r_0799 | nucleoside diphosphate kinase | ATP[c] + dTDP[c] + H+[c] => ADP[c] + dTTP[c] | 2.7.4.6 | PAPLA_01099 or PAPLA_04870 |
| r_0800 | nucleoside diphosphate kinase | ATP[c] + GDP[c] + H+[c] => ADP[c] + GTP[c] | 2.7.4.6 | PAPLA_01099 or PAPLA_04870 |
| r_0802 | nucleoside diphosphate kinase | ATP[c] + dIDP[c] <=> ADP[c] + dITP[c] + 3 H+[c] | 2.7.4.6 | PAPLA_01099 or PAPLA_04870 |
| r_0803 | nucleoside diphosphate kinase | ATP[c] + dUDP[c] + H+[c] <=> ADP[c] + dUTP[c] | 2.7.4.6 | PAPLA_01099 or PAPLA_04870 |
| r_0804 | nucleoside triphosphatase | GTP[c] + H2O[c] => GDP[c] + H+[c] + phosphate[c] + H+[c] | 3.6.1.5 | PAPLA_00471 or PAPLA_00483 or PAPLA_00760 or PAPLA_03664 or PAPLA_05894 |
| r_0813 | O-acetylhomoserine (thiol)-lyase | hydrogen sulfide[c] + O-acetyl-L-homoserine[c] + H+[c] => acetate[c] + H+[c] + L-homocysteine[c] | 2.5.1.47;2.5.1.49 | PAPLA_05226 |
| r_0831 | oxoglutarate dehydrogenase (dihydrolipoamide S-succinyltransferase) | coenzyme A[m] + S(8)-succinyldihydrolipoamide[m] => dihydrolipoamide[m] + succinyl-CoA[m] + H+[m] | 1.2.4.2;1.8.1.4;2.3.1.61 | (PAPLA_03685 and PAPLA_02484 and PAPLA_01932) or (PAPLA_03685 and PAPLA_02484 and PAPLA_03721) |
| r_0832 | oxoglutarate dehydrogenase (lipoamide) | 2-oxoglutarate[m] + H+[m] + lipoamide[m] => carbon dioxide[m] + S(8)-succinyldihydrolipoamide[m] + H+[m] | 1.2.4.2;1.8.1.4;2.3.1.61 | (PAPLA_03685 and PAPLA_02484 and PAPLA_01932) or (PAPLA_03685 and PAPLA_02484 and PAPLA_03721) |
| r_0842 | pantothenate kinase | (R)-pantothenate[c] + ATP[c] + 3 H+[c] => (R)-4'-phosphopantothenic acid[c] + ADP[c] + H+[c] | 2.7.1.33 | PAPLA_02884 |
| r_0852 | phenylalanyl-tRNA synthetase | ATP[c] + L-phenylalanine[c] + tRNA(Phe)[c] => AMP[c] + diphosphate[c] + Phe-tRNA(Phe)[c] | 6.1.1.20 | PAPLA_01355 and PAPLA_00912 |
| r_0853 | phenylalanyl-tRNA synthetase | ATP[m] + L-phenylalanine[m] + tRNA(Phe)[m] => AMP[m] + diphosphate[m] + Phe-tRNA(Phe)[m] | 6.1.1.20 | PAPLA_00649 |
| r_0855 | phopshoribosylaminoimidazole synthetase | 5'-phosphoribosyl-N-formylglycineamidine[c] + ATP[c] + H+[c] => 5'-phosphoribosyl-5-aminoimidazole[c] + ADP[c] + 2 H+[c] + phosphate[c] | 6.3.3.1;6.3.4.13 | PAPLA_03248 |
| r_0882 | phosphoacetylglucosamine mutase | N-acetyl-D-glucosamine 6-phosphate[c] +2 H+[c] <=> N-acetyl-alpha-D-glucosamine 1-phosphate[c] | 5.4.2.3 | PAPLA_05097 |
| r_0883 | phosphoadenylyl-sulfate reductase (thioredoxin) | 3'-phospho-5'-adenylyl sulfate[c] + TRX1[c] => adenosine 3',5'-bismonophosphate[c] + H+[c] + sulphite[c] + TRX1 disulphide[c] | 1.8.4.8 | PAPLA_04953 and PAPLA_03829 |
| r_0886 | phosphofructokinase | ATP[c] + D-fructose 6-phosphate[c] + 4 H+[c] => ADP[c] + D-fructose 1,6-bisphosphate[c] + H+[c] | 2.7.1.11 | PAPLA_01164 |
| r_0888 | phosphoglucomutase | D-glucose 6-phosphate[c] <=> D-glucose 1-phosphate[c] + 2 H+[c] | 5.4.2.2 |  |
| r_0890 | phosphoglucosamine mutase | alpha-D-glucosamine 1-phosphate[c] + H+[c] <=> alpha-D-glucosamine 6-phosphate[c] | 5.4.2.3 | PAPLA_05097 |
| r_0893 | phosphoglycerate mutase | 3-phosphonato-D-glycerate(3-)[c] <=> 2-phospho-D-glyceric acid[c] | 5.4.2.11 | PAPLA_02614 |
| r_0905 | phosphopantothenate-cysteine ligase | (R)-4'-phosphopantothenic acid[c] + CTP[c] + L-cysteine[c] => CMP[c] + diphosphate[c] + H+[c] | 6.3.2.5 | PAPLA_00204 |
| r_0906 | phosphopantothenoylcysteine decarboxylase | H+[c] => carbon dioxide[c] + pantetheine 4'-phosphate[c] | 4.1.1.36 | PAPLA_04439 |
| r_0907 | phosphopentomutase | alpha-D-ribose 1-phosphate(2-)[c] + 2 H+[c] <=> ribose-5-phosphate[c] | 5.4.2.2;5.4.2.7 |  |
| r_0911 | phosphoribosylaminoimidazole-carboxylase | 5'-phosphoribosyl-5-aminoimidazole[c] + ATP[c] + carbon dioxide[c] + H2O[c] => ADP[c] + 3 H+[c] + phosphate[c] + phosphoribosyl-carboxy-aminoimidazole[c] | 4.1.1.21 | PAPLA_04906 |
| r_0912 | phosphoribosylaminoimidazolecarboxamide formyltransferase | 10-formyl-THF[c] + AICAR[c] + 2 H+[c] <=> phosphoribosyl-formamido-carboxamide[c] + THF[c] | 2.1.2.3;3.5.4.10 | PAPLA_05237 |
| r_0916 | phosphoribosylpyrophosphate synthetase | ATP[c] + ribose-5-phosphate[c] => AMP[c] + 3 H+[c] + PRPP[c] | 2.7.6.1 | PAPLA_00298 or PAPLA_00871 |
| r_0921 | Phytosphingosine phosphate lyase | phytosphingosine 1-phosphate[er] => 2-hydroxyhexadecanal[er] + O-phosphoethanolamine[er] + H+[er] | 4.1.2.27 | PAPLA_00864 |
| r_0922 | phytosphingosine synthesis | H+[er] + NADPH[er] + oxygen[er] + sphinganine[er] => H2O[er] + NADP(+)[er] + phytosphingosine[er] + H+[er] | 1.-.-.- | PAPLA_03797 |
| r_0941 | prolyl-tRNA synthetase | ATP[c] + L-proline[c] + tRNA(Pro)[c] => AMP[c] + diphosphate[c] + H+[c] + Pro-tRNA(Pro)[c] | 6.1.1.15 | PAPLA_04093 |
| r_0967 | riboflavin synthase | 2-hydroxy-3-oxobutyl phosphate[c] + 5-amino-6-(D-ribitylamino)uracil[c] => 6,7-dimethyl-8-(1-D-ribityl)lumazine[c] + 2 H2O[c] + phosphate[c] + 3 H+[c] | 2.5.1.78 | PAPLA_05801 |
| r_0968 | riboflavin synthase | 2 6,7-dimethyl-8-(1-D-ribityl)lumazine[c] + H+[c] => 5-amino-6-(D-ribitylamino)uracil[c] + riboflavin[c] | 2.5.1.9 | PAPLA_00955 |
| r_0974 | ribonucleotide reductase | ADP[c] + TRX1[c] => dADP[c] + H2O[c] + TRX1 disulphide[c] | 1.17.4.1 | PAPLA_00211 or PAPLA_01944 or PAPLA_02335 |
| r_0975 | ribonucleotide reductase | ADP[n] + TRX1[n] => dADP[n] + H2O[n] + TRX1 disulphide[n] | 1.17.4.1 | PAPLA_00211 or PAPLA_01944 or PAPLA_02335 |
| r_0976 | ribonucleotide reductase | CDP[c] + TRX1[c] => dCDP[c] + H2O[c] + TRX1 disulphide[c] | 1.17.4.1 | PAPLA_00211 or PAPLA_01944 or PAPLA_02335 |
| r_0977 | ribonucleotide reductase | CDP[n] + TRX1[n] => dCDP[n] + H2O[n] + TRX1 disulphide[n] | 1.17.4.1 | PAPLA_00211 or PAPLA_01944 or PAPLA_02335 |
| r_0978 | ribonucleotide reductase | GDP[c] + TRX1[c] => dGDP[c] + H2O[c] + TRX1 disulphide[c] | 1.17.4.1 | PAPLA_00211 or PAPLA_01944 or PAPLA_02335 |
| r_0979 | ribonucleotide reductase | GDP[n] + TRX1[n] => dGDP[n] + H2O[n] + TRX1 disulphide[n] | 1.17.4.1 | PAPLA_00211 or PAPLA_01944 or PAPLA_02335 |
| r_0982 | ribose-5-phosphate isomerase | D-ribulose 5-phosphate[c] + 2 H+[c] <=> ribose-5-phosphate[c] | 5.3.1.6 | PAPLA_02672 |
| r_0985 | S-adenosyl-L-methionine:3-hexaprenyl-4,5-dihydroxylate O-methyltransferase | 3-hexaprenyl-4,5-dihydroxybenzoic acid[m] + S-adenosyl-L-methionine[m] => 3-hexaprenyl-4-hydroxy-5-methoxybenzoic acid[m] + H+[m] + S-adenosyl-L-homocysteine[m] | 1.14.13.-;2.1.1.114;2.1.1.201;2.1.1.64;2.7.-.- | (PAPLA_02366 and PAPLA_05126 and PAPLA_06261 and PAPLA_02748 and PAPLA_04581 and PAPLA_04074) or (PAPLA_02366 and PAPLA_05126 and PAPLA_06261 and PAPLA_04409 and PAPLA_04581 and PAPLA_04074) |
| r_0993 | serine palmitotransferase | L-serine[er] + palmitoyl-CoA[er] + H+[c] => 3-ketosphinganine[er] + carbon dioxide[er] + coenzyme A[er] | 2.3.1.50 | PAPLA_00672 and PAPLA_05286 |
| r_0995 | seryl-tRNA synthetase | ATP[c] + L-serine[c] + tRNA(Ser)[c] => AMP[c] + diphosphate[c] + Ser-tRNA(Ser)[c] | 6.1.1.11 | PAPLA_02705 or PAPLA_03572 |
| r_0998 | sirohydrochlorin dehydrogenase | NAD[c] + precorrin-2[c] => 2 H+[c] + NADH[c] + sirohydrochlorin[c] | 1.3.1.76;4.99.1.4 | PAPLA_05908 |
| r_0999 | sirohydrochlorin ferrochetalase | iron(2+)[c] + sirohydrochlorin[c] + H+[c] => 3 H+[c] + siroheme[c] | 1.3.1.76;4.99.1.4 | PAPLA_05908 |
| r_1004 | sphingoid base-phosphate phosphatase (phytosphingosine 1-phosphate) | H2O[er] + phytosphingosine 1-phosphate[er] => phosphate[er] + phytosphingosine[er] + 2 H+[er] | 3.1.3.- | PAPLA_04799 |
| r_1027 | sulfite reductase (NADPH2) | 5 H+[c] + 3 NADPH[c] + sulphite[c] => 3 H2O[c] + hydrogen sulfide[c] + 3 NADP(+)[c] + 2 H+[c] | 1.8.1.2 | PAPLA_04979 and PAPLA_00807 |
| r_1029 | taurine dioxygenase | 2-oxoglutarate[c] + oxygen[c] + taurine[c] + H+[c]=> aminoacetaldehyde[c] + carbon dioxide[c] + H+[c] + succinate[c] + sulphite[c] | 1.14.11.- | PAPLA_00018 or PAPLA_00355 or PAPLA_00800 or PAPLA_06369 |
| r_1031 | tetrahydrofolate:L-glutamate gamma-ligase (ADP-forming) | ATP[c] + L-glutamate[c] + THF[c] + H+[c] <=> 5,6,7,8-tetrahydrofolyl-L-glutamic acid[c] + ADP[c] + H+[c] + phosphate[c] | 6.3.2.17 | PAPLA_01919 or PAPLA_04187 |
| r_1032 | thiamin diphosphatase | 2 H2O[e] + TDP[e] => 3 H+[e] + 2 phosphate[e] + thiamine[e] | 3.1.3.2 | PAPLA_02832 or PAPLA_04156 |
| r_1033 | thiamin phosphatase | H2O[e] + TMP[e] => phosphate[e] + thiamine[e] + 2 H+[e] | 3.1.3.2 | PAPLA_02832 or PAPLA_04156 |
| r_1034 | thiamine diphosphokinase | ATP[c] + thiamine[c] + 2 H+[c]=> AMP[c] + H+[c] + TDP[c] | 2.7.6.2 | PAPLA_01558 |
| r_1035 | thiamine-diphosphate kinase | ATP[c] + TDP[c] => ADP[c] + thiamine(1+) triphosphate(4-)[c] + 2 H+[c] | 2.7.6.2 | PAPLA_01558 |
| r_1038 | thioredoxin reductase (NADPH) | H+[c] + NADPH[c] + TRX1 disulphide[c] => NADP(+)[c] + TRX1[c] | 1.8.1.9 | PAPLA_05920 |
| r_1039 | thioredoxin reductase (NADPH) | H+[m] + NADPH[m] + TRX1 disulphide[m] => NADP(+)[m] + TRX1[m] | 1.8.1.7;1.8.1.9 | PAPLA_05035 or PAPLA_05920 |
| r_1042 | threonyl-tRNA synthetase | ATP[c] + L-threonine[c] + tRNA(Thr)[c] => AMP[c] + diphosphate[c] + Thr-tRNA(Thr)[c] | 6.1.1.3 | PAPLA_03608 |
| r_1043 | threonyl-tRNA synthetase | ATP[m] + L-threonine[m] + tRNA(Thr)[m] => AMP[m] + diphosphate[m] + Thr-tRNA(Thr)[m] | 6.1.1.3 | PAPLA_03608 |
| r_1045 | thymidylate synthase | 5,10-methylenetetrahydrofolate[c] + dUMP[c] + 2 H+[c] => dihydrofolic acid[c] + dTMP[c] | 2.1.1.45 | PAPLA_01200 |
| r_1049 | transketolase 1 | D-xylulose 5-phosphate[c] + ribose-5-phosphate[c] <=> glyceraldehyde 3-phosphate[c] + sedoheptulose 7-phosphate[c] + 2 H+[c] | 2.2.1.1 | PAPLA_01204 or PAPLA_02392 or PAPLA_05246 |
| r_1051 | trehalose-phosphatase | alpha,alpha-trehalose 6-phosphate[c] + H2O[c] => phosphate[c] + trehalose[c] | 2.4.1.15;3.1.3.12 | PAPLA_01725 or PAPLA_03918 |
| r_1057 | tryptophanyl-tRNA synthetase | ATP[c] + L-tryptophan[c] + tRNA(Trp)[c] => AMP[c] + diphosphate[c] + Trp-tRNA(Trp)[c] | 6.1.1.2 | PAPLA_01118 |
| r_1066 | tyrosyl-tRNA synthetase | ATP[c] + L-tyrosine[c] + tRNA(Tyr)[c] => AMP[c] + diphosphate[c] + Tyr-tRNA(Tyr)[c] | 6.1.1.1 | PAPLA_04574 |
| r_1067 | tyrosyl-tRNA synthetase | ATP[m] + L-tyrosine[m] + tRNA(Tyr)[m] => AMP[m] + diphosphate[m] + Tyr-tRNA(Tyr)[m] | 6.1.1.1 | PAPLA_04072 |
| r_1069 | UDP-N-acetylglucosamine diphosphorylase | H+[c] + N-acetyl-alpha-D-glucosamine 1-phosphate[c] + UTP[c] <=> diphosphate[c] + UDP-N-acetyl-alpha-D-glucosamine[c] + 2 H+[c] | 2.7.7.23 | PAPLA_05912 |
| r_1076 | ureidoglycolate hydrolase | 2 H+[c] + H2O[c] + ureidoglycolic acid[c] => 2 ammonium[c] + carbon dioxide[c] + glyoxylate[c] + H+[c] | 4.3.2.3 | PAPLA_02905 |
| r_1078 | uridine kinase (GTP:uridine) | GTP[c] + uridine[c] => GDP[c] + 2 H+[c] + UMP[c] | 2.7.1.48 | PAPLA_00418 |
| r_1089 | valyl-tRNA synthetase | ATP[c] + L-valine[c] + tRNA(Val)[c] => AMP[c] + diphosphate[c] + Val-tRNA(Val)[c] | 6.1.1.9 | PAPLA_03404 |
| r_1090 | valyl-tRNA synthetase | ATP[m] + L-valine[m] + tRNA(Val)[m] => AMP[m] + diphosphate[m] + Val-tRNA(Val)[m] | 6.1.1.9 | PAPLA_03404 |
| r_1095 | yUMP synthetase | ribose-5-phosphate[c] + uracil[c] <=> H2O[c] + pseudouridine 5'-phosphate[c] + 2 H+[c] | 5.4.99.-;5.4.99.25;5.4.99.44;5.4.99.45 | PAPLA_00527 or PAPLA_03458 |
| r_2112 | kynurenine aminotransferase | L-kynurenine[c] + pyruvate[c] + H+[c] <=> L-alanine[c] + kynurenic acid[c] + H2O[c] | 2.6.1.7 | PAPLA_04670 |
| r_2116 | acetaldehyde dehydrogenase | acetaldehyde[c] + H2O[c] + NAD[c] => acetate[c] + 2 H+[c] + NADH[c] | 1.2.1.3 | PAPLA_00036 or PAPLA_01760 or PAPLA_02435 or PAPLA_03515 or PAPLA_04152 or PAPLA_04722 or PAPLA_05667 |
| r_2126 | sedoheptulose bisphosphatase | sedoheptulose 1,7-bisphosphate[c] + H2O => phosphate[c] + sedoheptulose 7-phosphate[c] | 3.1.3.37 | PAPLA_05868 |
| r_2168 | B-hydroxyacyl-CoA dehydratase (trans-tetradec-2-enoyl-CoA) | (S)-3-hydroxytetradecanoyl-CoA[erm] <=> H2O[erm] + trans-tetradec-2-enoyl-CoA[erm] + 4 H+[erm] | 4.2.1.134 | PAPLA_05164 |
| r_2171 | B-hydroxyacyl-CoA dehydratase (trans-icos-2-enoyl-CoA) | 3-hydroxyicosanoyl-CoA[erm] <=> H2O[erm] + trans-icos-2-enoyl-CoA[erm] | 4.2.1.134 | PAPLA_05164 |
| r_2172 | B-hydroxyacyl-CoA dehydratase (trans-docos-2-enoyl-CoA) | 3-hydroxydocosanoyl-CoA[erm] <=> H2O[erm] + trans-docos-2-enoyl-CoA[erm] | 4.2.1.134 | PAPLA_05164 |
| r_2176 | trans-2-enoyl-CoA reductase (n-C16:0CoA) | H+[erm] + NADPH[erm] + trans-hexadec-2-enoyl-CoA[erm] => palmitoyl-CoA[erm] + NADP(+)[erm] + 4 H+[erm] | 1.3.1.93 | PAPLA_04901 |
| r_2177 | trans-2-enoyl-CoA reductase (n-C18:0CoA) | H+[erm] + NADPH[erm] + trans-octadec-2-enoyl-CoA[erm] => stearoyl-CoA[erm] + NADP(+)[erm] + 4 H+[erm] | 1.3.1.93 | PAPLA_04901 |
| r_2178 | trans-2-enoyl-CoA reductase (n-C20:0CoA) | H+[erm] + NADPH[erm] + trans-icos-2-enoyl-CoA[erm] => icosanoyl-CoA[erm] + NADP(+)[erm] | 1.3.1.93 | PAPLA_04901 |
| r_2179 | trans-2-enoyl-CoA reductase (n-C22:0CoA) | H+[erm] + NADPH[erm] + trans-docos-2-enoyl-CoA[erm] => docosanoyl-CoA[erm] + NADP(+)[erm] | 1.3.1.93 | PAPLA_04901 |
| r_2238 | acyl-CoA oxidase (octanoyl-CoA) | octanoyl-CoA[p] + oxygen[p] + 4 H+[p] => hydrogen peroxide[p] + trans-oct-2-enoyl-CoA[p] | 1.3.3.6 | PAPLA_02012 or PAPLA_02013 |
| r_2239 | acyl-CoA oxidase (icosanoyl-CoA) | oxygen[p] + icosanoyl-CoA[p] => hydrogen peroxide[p] + trans-icos-2-enoyl-CoA[p] | 1.3.3.6 | PAPLA_02012 or PAPLA_02013 |
| r_2240 | acyl-CoA oxidase (docosanoyl-CoA) | oxygen[p] + docosanoyl-CoA[p] => hydrogen peroxide[p] + trans-docos-2-enoyl-CoA[p] | 1.3.3.6 | PAPLA_02012 or PAPLA_02013 |
| r_2242 | acyl-CoA oxidase (palmitoleoyl-CoA) | oxygen[p] + palmitoleoyl-CoA(4-)[p] + 4 H+[p] => hydrogen peroxide[p] + trans-2,cis-9-hexadecadienoyl-CoA[p] | 1.3.3.6 | PAPLA_02012 or PAPLA_02013 |
| r_2244 | acyl-CoA oxidase (cis-dodec-5-enoyl-CoA) | oxygen[p] + cis-dodec-5-enoyl-CoA[p] => hydrogen peroxide[p] + trans-2,cis-5-dodecadienoyl-CoA[p] | 1.3.3.6 | PAPLA_02012 or PAPLA_02013 |
| r_2245 | acyl-CoA oxidase (oleoyl-CoA) | oleoyl-CoA[p] + oxygen[p] => hydrogen peroxide[p] + trans-2,cis-9-octadecadienoyl-CoA[p] | 1.3.3.6 | PAPLA_02012 or PAPLA_02013 |
| r_2248 | 2-enoyl-CoA hydratase (3-hydroxydecanoyl-CoA) | H2O[p] + trans-dec-2-enoyl-CoA[p] + 4 H+[p] => (R)-3-hydroxydecanoyl-CoA[p] | 1.1.1.n12;4.2.1.119 | PAPLA_04129 or PAPLA_04130 |
| r_2249 | 2-enoyl-CoA hydratase (3-hydroxydodecanoyl-CoA) | H2O[p] + trans-dodec-2-enoyl-CoA[p] + 4 H+[p] => (R)-3-hydroxylauroyl-CoA[p] | 1.1.1.n12;4.2.1.119 | PAPLA_04129 or PAPLA_04130 |
| r_2250 | 2-enoyl-CoA hydratase (3-hydroxytetradecanoyl-CoA) | H2O[p] + trans-tetradec-2-enoyl-CoA[p] + 4 H+[p] => (S)-3-hydroxytetradecanoyl-CoA[p] | 1.1.1.n12;4.2.1.119 | PAPLA_04129 or PAPLA_04130 |
| r_2256 | 2-enoyl-CoA hydratase (3-hydroxyoctanoyl-CoA) | H2O[p] + trans-oct-2-enoyl-CoA[p] => (R)-3-hydroxyoctanoyl-CoA[p] | 1.1.1.n12;4.2.1.119 | PAPLA_04129 or PAPLA_04130 |
| r_2257 | 2-enoyl-CoA hydratase (3-hydroxyicosanoyl-CoA) | H2O[p] + trans-icos-2-enoyl-CoA[p] => (R)-3-hydroxyicosanoyl-CoA[p] | 1.1.1.n12;4.2.1.119 | PAPLA_04129 or PAPLA_04130 |
| r_2258 | 2-enoyl-CoA hydratase (3-hydroxydocosanoyl-CoA) | H2O[p] + trans-docos-2-enoyl-CoA[p] => (R)-3-hydroxydocosanoyl-CoA[p] | 1.1.1.n12;4.2.1.119 | PAPLA_04129 or PAPLA_04130 |
| r_2261 | 2-enoyl-CoA hydratase (3-hydroxy-cis-tetradec-7-enoyl-CoA) | H2O[p] + trans-2,cis-7-tetradecadienoyl-CoA[p] => (R)-3-hydroxy-cis-tetradec-7-enoyl-CoA[p] | 1.1.1.n12;4.2.1.119 | PAPLA_04129 or PAPLA_04130 |
| r_2262 | 2-enoyl-CoA hydratase (3-hydroxy-cis-dodec-5-enoyl-CoA) | H2O[p] + trans-2,cis-5-dodecadienoyl-CoA[p] => (R)-3-hydroxy-cis-dodec-5-enoyl-CoA[p] | 1.1.1.n12;4.2.1.119 | PAPLA_04129 or PAPLA_04130 |
| r_2263 | 2-enoyl-CoA hydratase (3-hydroxy-cis-octadec-9-enoyl-CoA) | H2O[p] + trans-2,cis-9-octadecadienoyl-CoA[p] + 4 H+[p]=> (R)-3-hydroxy-cis-octadec-9-enoyl-CoA[p] | 1.1.1.n12;4.2.1.119 | PAPLA_04129 or PAPLA_04130 |
| r_2268 | 3-hydroxyacyl-CoA dehydrogenase (3-oxohexadecanoyl-CoA) | (S)-3-hydroxypalmitoyl-CoA[p] + NAD[p] => 3-oxopalmitoyl-CoA[p] + 5 H+[p] + NADH[p] | 1.1.1.n12;4.2.1.119 | PAPLA_04129 or PAPLA_04130 |
| r_2271 | 3-hydroxyacyl-CoA dehydrogenase (3-oxobutanoyl-CoA) | NAD[p] + (R)-3-hydroxybutanoyl-CoA[p] => 5 H+[p] + NADH[p] + acetoacetyl-CoA[p] | 1.1.1.n12;4.2.1.119 | PAPLA_04129 or PAPLA_04130 |
| r_2273 | 3-hydroxyacyl-CoA dehydrogenase (3-oxooctanoyl-CoA) | NAD[p] + (R)-3-hydroxyoctanoyl-CoA[p] => H+[p] + NADH[p] + 3-oxooctanoyl-CoA[p] | 1.1.1.n12;4.2.1.119 | PAPLA_04129 or PAPLA_04130 |
| r_2275 | 3-hydroxyacyl-CoA dehydrogenase (3-oxodocosanoyl-CoA) | NAD[p] + (R)-3-hydroxydocosanoyl-CoA[p] + 4 H+[p] => H+[p] + NADH[p] + 3-oxodocosanoyl-CoA[p] | 1.1.1.n12;4.2.1.119 | PAPLA_04129 or PAPLA_04130 |
| r_2277 | 3-hydroxyacyl-CoA dehydrogenase (3-oxo-cis-hexadec-9-enoyl-CoA) | NAD[p] + (R)-3-hydroxy-cis-hexadec-9-enoyl-CoA[p] => H+[p] + NADH[p] + 3-oxo-cis-hexadec-9-enoyl-CoA[p] | 1.1.1.n12;4.2.1.119 | PAPLA_04129 or PAPLA_04130 |
| r_2278 | 3-hydroxyacyl-CoA dehydrogenase (3-oxo-cis-tetradec-7-enoyl-CoA) | NAD[p] + (R)-3-hydroxy-cis-tetradec-7-enoyl-CoA[p] => H+[p] + NADH[p] + 3-oxo-cis-tetradec-7-enoyl-CoA[p] | 1.1.1.n12;4.2.1.119 | PAPLA_04129 or PAPLA_04130 |
| r_2279 | 3-hydroxyacyl-CoA dehydrogenase (3-oxo-cis-dodec-5-enoyl-CoA) | NAD[p] + (R)-3-hydroxy-cis-dodec-5-enoyl-CoA[p] => H+[p] + NADH[p] + 3-oxo-cis-dodec-5-enoyl-CoA[p] | 1.1.1.n12;4.2.1.119 | PAPLA_04129 or PAPLA_04130 |
| r_2280 | 3-hydroxyacyl-CoA dehydrogenase (3-oxo-cis-octadec-9-enoyl-CoA) | NAD[p] + (R)-3-hydroxy-cis-octadec-9-enoyl-CoA[p] => H+[p] + NADH[p] + 3-oxo-cis-octadec-9-enoyl-CoA[p] | 1.1.1.n12;4.2.1.119 | PAPLA_04129 or PAPLA_04130 |
| r_2281 | 3-hydroxyacyl-CoA dehydrogenase (3-oxo-cis-hexadec-7-enoyl-CoA) | NAD[p] + (R)-3-hydroxy-cis-hexadec-7-enoyl-CoA[p] => H+[p] + NADH[p] + 3-oxo-cis-hexadec-7-enoyl-CoA[p] | 1.1.1.n12;4.2.1.119 | PAPLA_04129 or PAPLA_04130 |
| r_2282 | 3-hydroxyacyl-CoA dehydrogenase (3-oxo-cis-tetradec-5-enoyl-CoA) | NAD[p] + (R)-3-hydroxy-cis-tetradec-5-enoyl-CoA[p] => H+[p] + NADH[p] + 3-oxo-cis-tetradec-5-enoyl-CoA[p] | 1.1.1.n12;4.2.1.119 | PAPLA_04129 or PAPLA_04130 |
| r_2286 | acetyl-CoA C-acyltransferase (stearoyl-CoA) | coenzyme A[p] + 3-oxoicosanoyl-CoA[p] => acetyl-CoA[p] + stearoyl-CoA[p] + 4 H+[p] | 2.3.1.16 | PAPLA_00899 or PAPLA_01492 or PAPLA_01644 |
| r_2289 | acetyl-CoA C-acyltransferase (cis-tetradec-7-enoyl-CoA) | coenzyme A[p] + 3-oxo-cis-hexadec-9-enoyl-CoA[p] => acetyl-CoA[p] + cis-tetradec-7-enoyl-CoA[p] | 2.3.1.16 | PAPLA_00899 or PAPLA_01492 or PAPLA_01644 |
| r_2290 | acetyl-CoA C-acyltransferase (cis-dodec-5-enoyl-CoA) | coenzyme A[p] + 3-oxo-cis-tetradec-7-enoyl-CoA[p] => acetyl-CoA[p] + cis-dodec-5-enoyl-CoA[p] | 2.3.1.16 | PAPLA_00899 or PAPLA_01492 or PAPLA_01644 |
| r_2291 | acetyl-CoA C-acyltransferase (cis-dec-3-enoyl-CoA) | coenzyme A[p] + 3-oxo-cis-dodec-5-enoyl-CoA[p] => acetyl-CoA[p] + cis-dec-3-enoyl-CoA[p] | 2.3.1.16 | PAPLA_00899 or PAPLA_01492 or PAPLA_01644 |
| r_2292 | acetyl-CoA C-acyltransferase (cis-hexadec-7-enoyl-CoA) | coenzyme A[p] + 3-oxo-cis-octadec-9-enoyl-CoA[p] => acetyl-CoA[p] + cis-hexadec-7-enoyl-CoA[p] | 2.3.1.16 | PAPLA_00899 or PAPLA_01492 or PAPLA_01644 |
| r_2293 | acetyl-CoA C-acyltransferase (cis-tetradec-5-enoyl-CoA) | coenzyme A[p] + 3-oxo-cis-hexadec-7-enoyl-CoA[p] => acetyl-CoA[p] + cis-tetradec-5-enoyl-CoA[p] | 2.3.1.16 | PAPLA_00899 or PAPLA_01492 or PAPLA_01644 |
| r_2294 | acetyl-CoA C-acyltransferase (cis-dodec-3-enoyl-CoA) | coenzyme A[p] + 3-oxo-cis-tetradec-5-enoyl-CoA[p] => acetyl-CoA[p] + cis-dodec-3-enoyl-CoA[p] | 2.3.1.16 | PAPLA_00899 or PAPLA_01492 or PAPLA_01644 |
| r_2304 | 2,4-dienoyl-CoA reductase (trans-2,trans-4-tetradecadienoyl-CoA) | H+[p] + NADPH[p] + trans-2,trans-4-tetradecadienoyl-CoA[p] => NADP(+)[p] + trans-tetradec-3-enoyl-CoA[p] | 1.3.1.34 | PAPLA_06105 |
| r_2316 | glycerol-3-phosphate acyltransferase (16:0), lipid particle | glycerol 3-phosphate[lp] + palmitoyl-CoA[lp] + 2 H+[lp] => coenzyme A[lp] + 1-acyl-sn-glycerol 3-phosphate (16:0)[lp] |  |  |
| r_2318 | glycerol-3-phosphate acyltransferase (18:0), lipid particle | glycerol 3-phosphate[lp] + stearoyl-CoA[lp] + 2 H+[lp] => coenzyme A[lp] + 1-acyl-sn-glycerol 3-phosphate (18:0)[lp] |  |  |
| r_2320 | dihydroxyacetone phosphate acyltransferase (16:0), lipid particle | dihydroxyacetone phosphate[lp] + palmitoyl-CoA[lp] + 2 H+[lp] => acylglycerone phosphate (16:0)[lp] + coenzyme A[lp] |  |  |
| r_2330 | acyl dhap reductase (18:0), lipid particle | acylglycerone phosphate (18:0)[lp] + 3 H+[lp] + NADPH[lp] => 1-acyl-sn-glycerol 3-phosphate (18:0)[lp] + NADP(+)[lp] |  |  |
| r_2353 | PA phosphatase (1-16:0, 2-18:1), vacuolar membrane | H2O[vm] + phosphatidate (1-16:0, 2-18:1)[vm] => phosphate[vm] + diglyceride (1-16:0, 2-18:1)[vm] + 2 H+[vm] |  | PAPLA_05340 |
| r_2357 | PA phosphatase (1-18:0, 2-18:1), vacuolar membrane | H2O[vm] + phosphatidate (1-18:0, 2-18:1)[vm] => phosphate[vm] + diglyceride (1-18:0, 2-18:1)[vm] + 2 H+[vm] |  | PAPLA_05340 |
| r_2468 | PS decarboxylase (1-16:0, 2-18:1), mitochondrial membrane | H+[mm] + phosphatidyl-L-serine (1-16:0, 2-18:1)[mm] => carbon dioxide[mm] + phosphatidylethanolamine (1-16:0, 2-18:1)[mm] + H+[mm] |  | PAPLA_00279 |
| r_2470 | PS decarboxylase (1-18:0, 2-18:1), mitochondrial membrane | H+[mm] + phosphatidyl-L-serine (1-18:0, 2-18:1)[mm] => carbon dioxide[mm] + phosphatidylethanolamine (1-18:0, 2-18:1)[mm] + H+[mm] |  | PAPLA_00279 |
| r_2484 | PS decarboxylase (1-16:0, 2-18:1), vacuolar membrane | H+[vm] + phosphatidyl-L-serine (1-16:0, 2-18:1)[vm] => carbon dioxide[vm] + phosphatidylethanolamine (1-16:0, 2-18:1)[vm] + H+[vm] |  | PAPLA_01262 or PAPLA_02654 |
| r_2486 | PS decarboxylase (1-18:0, 2-18:1), vacuolar membrane | H+[vm] + phosphatidyl-L-serine (1-18:0, 2-18:1)[vm] => carbon dioxide[vm] + phosphatidylethanolamine (1-18:0, 2-18:1)[vm] + H+[vm] |  | PAPLA_01262 or PAPLA_02654 |
| r_2540 | phosphatidylglycerolphosphate synthase (1-16:0, 2-18:1), mitochondrial membrane | CDP-diacylglycerol (1-16:0, 2-18:1)[mm] + glycerol 3-phosphate[mm] => 2 H+[mm] + CMP[mm] + 3-(3-sn-phosphatidyl)-sn-glycerol 1-phosphate (1-16:0, 2-18:1)[mm] |  | PAPLA_03226 |
| r_2546 | PGP phosphatase (1-16:0, 2-18:1), mitochondrial membrane | 3-(3-sn-phosphatidyl)-sn-glycerol 1-phosphate (1-16:0, 2-18:1)[mm] + H2O[mm] => phosphate[mm] + phosphatidylglycerol (1-16:0, 2-18:1)[mm] |  | PAPLA_01052 |
| r_2576 | CL synthase (1-16:0, 2-18:1, 3-16:0, 4-18:1), mitochondrial membrane | CDP-diacylglycerol (1-16:0, 2-18:1)[mm] + phosphatidylglycerol (1-16:0, 2-18:1)[mm] => 2 H+[mm] + CMP[mm] + cardiolipin (1-16:0, 2-18:1, 3-16:0, 4-18:1)[mm] |  | PAPLA_03081 |
| r_2600 | CL (1-16:0, 2-18:1, 3-16:0, 4-18:1) phospholipase (1-position), mitochondrial membrane | H2O[mm] + cardiolipin (1-16:0, 2-18:1, 3-16:0, 4-18:1)[mm] => H+[mm] + palmitate[mm] + monolysocardiolipin (2-18:1, 3-16:0, 4-18:1)[mm] |  |  |
| r_2704 | MLCL (2-18:1, 3-16:0, 4-18:1):PC (1-16:0, 2-18:1) acyltransferase, mitochondrial membrane | monolysocardiolipin (2-18:1, 3-16:0, 4-18:1)[mm] + phosphatidylcholine (1-16:0, 2-18:1)[mm] + 3 H+[mm] <=> 1-acylglycerophosphocholine (16:0)[mm] + cardiolipin (1-18:1, 2-18:1, 3-16:0, 4-18:1)[mm] |  | PAPLA_03750 |
| r_2813 | lysoPC acyltransferase (1-16:0, 2-18:1), mitochondrial membrane | 1-acylglycerophosphocholine (16:0)[mm] + oleoyl-CoA[mm] => phosphatidylcholine (1-16:0, 2-18:1)[mm] + coenzyme A[mm] + H+[mm] |  |  |
| r_2817 | lysoPC acyltransferase (1-18:0, 2-18:1), mitochondrial membrane | 1-acylglycerophosphocholine (18:0)[mm] + oleoyl-CoA[mm] => H+[mm] + phosphatidylcholine (1-18:0, 2-18:1)[mm] + coenzyme A[mm] |  |  |
| r_2824 | phosphatidylinositol 4-kinase (1-16:0, 2-18:1), cell envelope | ATP[ce] + 1-phosphatidyl-1D-myo-inositol (1-16:0, 2-18:1)[ce] + 2 H+[ce] => H+[ce] + ADP[ce] + 1-phosphatidyl-1D-myo-inositol 4-phosphate (1-16:0, 2-18:1)[ce] |  | PAPLA_02367 |
| r_2826 | phosphatidylinositol 4-kinase (1-18:0, 2-18:1), cell envelope | ATP[ce] + 1-phosphatidyl-1D-myo-inositol (1-18:0, 2-18:1)[ce] + 2 H+[ce] => H+[ce] + ADP[ce] + 1-phosphatidyl-1D-myo-inositol 4-phosphate (1-18:0, 2-18:1)[ce] |  | PAPLA_02367 |
| r_2832 | phosphatidylinositol 4-kinase (1-16:0, 2-18:1), vacuolar membrane | ATP[vm] + 1-phosphatidyl-1D-myo-inositol (1-16:0, 2-18:1)[vm] + 2 H+[vm] => 1-phosphatidyl-1D-myo-inositol 4-phosphate (1-16:0, 2-18:1)[vm] + H+[vm] + ADP[vm] |  |  |
| r_2834 | phosphatidylinositol 4-kinase (1-18:0, 2-18:1), vacuolar membrane | ATP[vm] + 1-phosphatidyl-1D-myo-inositol (1-18:0, 2-18:1)[vm] + 2 H+[vm] => 1-phosphatidyl-1D-myo-inositol 4-phosphate (1-18:0, 2-18:1)[vm] + H+[vm] + ADP[vm] |  |  |
| r_2840 | phosphatidylinositol 4-kinase (1-16:0, 2-18:1), Golgi membrane | ATP[gm] + 1-phosphatidyl-1D-myo-inositol (1-16:0, 2-18:1)[gm] + 2 H+[gm] => H+[gm] + ADP[gm] + 1-phosphatidyl-1D-myo-inositol 4-phosphate (1-16:0, 2-18:1)[gm] |  | (PAPLA_00428 and PAPLA_02367) or PAPLA_02367 |
| r_2842 | phosphatidylinositol 4-kinase (1-18:0, 2-18:1), Golgi membrane | ATP[gm] + 1-phosphatidyl-1D-myo-inositol (1-18:0, 2-18:1)[gm] + 2 H+[gm] => H+[gm] + ADP[gm] + 1-phosphatidyl-1D-myo-inositol 4-phosphate (1-18:0, 2-18:1)[gm] |  | (PAPLA_00428 and PAPLA_02367) or PAPLA_02367 |
| r_2848 | phosphatidylinositol 4-kinase (1-16:0, 2-18:1), nucleus | ATP[n] + 1-phosphatidyl-1D-myo-inositol (1-16:0, 2-18:1)[n] + 2 H+[n] => ADP[n] + H+[n] + 1-phosphatidyl-1D-myo-inositol 4-phosphate (1-16:0, 2-18:1)[n] |  | PAPLA_02367 |
| r_2850 | phosphatidylinositol 4-kinase (1-18:0, 2-18:1), nucleus | ATP[n] + 1-phosphatidyl-1D-myo-inositol (1-18:0, 2-18:1)[n] + 2 H+[n] => ADP[n] + H+[n] + 1-phosphatidyl-1D-myo-inositol 4-phosphate (1-18:0, 2-18:1)[n] |  | PAPLA_02367 |
| r_2856 | phosphatidylinositol 3-kinase (1-16:0, 2-18:1), vacuolar membrane | ATP[vm] + 1-phosphatidyl-1D-myo-inositol (1-16:0, 2-18:1)[vm] + 2 H+[vm] => H+[vm] + ADP[vm] + 1-phosphatidyl-1D-myo-inositol 3-phosphate (1-16:0, 2-18:1)[vm] |  | PAPLA_02262 and PAPLA_03964 |
| r_2858 | phosphatidylinositol 3-kinase (1-18:0, 2-18:1), vacuolar membrane | ATP[vm] + 1-phosphatidyl-1D-myo-inositol (1-18:0, 2-18:1)[vm] + 2 H+[vm] => H+[vm] + ADP[vm] + 1-phosphatidyl-1D-myo-inositol 3-phosphate (1-18:0, 2-18:1)[vm] |  | PAPLA_02262 and PAPLA_03964 |
| r_2864 | PI 4-P 5-kinase (1-16:0, 2-18:1), nucleus | ATP[n] + 1-phosphatidyl-1D-myo-inositol 4-phosphate (1-16:0, 2-18:1)[n] + 2 H+[n] => ADP[n] + H+[n] + 1-phosphatidyl-1D-myo-inositol 4,5-bisphosphate (1-16:0, 2-18:1)[n] |  | PAPLA_01574 |
| r_2866 | PI 4-P 5-kinase (1-18:0, 2-18:1), nucleus | ATP[n] + 1-phosphatidyl-1D-myo-inositol 4-phosphate (1-18:0, 2-18:1)[n] + 2 H+[n] => ADP[n] + H+[n] + 1-phosphatidyl-1D-myo-inositol 4,5-bisphosphate (1-18:0, 2-18:1)[n] |  | PAPLA_01574 |
| r_2872 | PI 4-P 5-kinase (1-16:0, 2-18:1), cell envelope | ATP[ce] + 1-phosphatidyl-1D-myo-inositol 4-phosphate (1-16:0, 2-18:1)[ce] + 2 H+[ce] => H+[ce] + ADP[ce] + 1-phosphatidyl-1D-myo-inositol 4,5-bisphosphate (1-16:0, 2-18:1)[ce] |  | PAPLA_01574 |
| r_2874 | PI 4-P 5-kinase (1-18:0, 2-18:1), cell envelope | ATP[ce] + 1-phosphatidyl-1D-myo-inositol 4-phosphate (1-18:0, 2-18:1)[ce] + 2 H+[ce] => H+[ce] + ADP[ce] + 1-phosphatidyl-1D-myo-inositol 4,5-bisphosphate (1-18:0, 2-18:1)[ce] |  | PAPLA_01574 |
| r_2880 | PI 3-P 5-kinase (1-16:0, 2-18:1), vacuolar membrane | ATP[vm] + 1-phosphatidyl-1D-myo-inositol 3-phosphate (1-16:0, 2-18:1)[vm] + 2 H+[vm] => H+[vm] + ADP[vm] + 1-phosphatidyl-1D-myo-inositol 3,5-bisphosphate (1-16:0, 2-18:1)[vm] |  | PAPLA_02729 or PAPLA_03129 |
| r_2882 | PI 3-P 5-kinase (1-18:0, 2-18:1), vacuolar membrane | ATP[vm] + 1-phosphatidyl-1D-myo-inositol 3-phosphate (1-18:0, 2-18:1)[vm] + 2 H+[vm] => H+[vm] + ADP[vm] + 1-phosphatidyl-1D-myo-inositol 3,5-bisphosphate (1-18:0, 2-18:1)[vm] |  | PAPLA_02729 or PAPLA_03129 |
| r_3038 | PC phospholipase B (1-16:0, 2-18:1), cell envelope | H2O[ce] + phosphatidylcholine (1-16:0, 2-18:1)[ce] + H+[ce] => 1-acylglycerophosphocholine (16:0)[ce] + H+[ce] + oleate[ce] |  | PAPLA_05497 |
| r_3040 | PC phospholipase B (1-18:0, 2-18:1), cell envelope | H2O[ce] + phosphatidylcholine (1-18:0, 2-18:1)[ce] + H+[ce] => 1-acylglycerophosphocholine (18:0)[ce] + H+[ce] + oleate[ce] |  | PAPLA_05497 |
| r_3042 | LPC phospholipase B (16:0), cell envelope | 1-acylglycerophosphocholine (16:0)[ce] + H2O[ce] => 2 H+[ce] + sn-glycero-3-phosphocholine[ce] + palmitate[ce] |  | PAPLA_05497 |
| r_3044 | LPC phospholipase B (18:0), cell envelope | 1-acylglycerophosphocholine (18:0)[ce] + H2O[ce] => 2 H+[ce] + sn-glycero-3-phosphocholine[ce] + stearate[ce] |  | PAPLA_05497 |
| r_3054 | LPE phospholipase B (16:0), cell envelope | 1-acylglycerophosphoethanolamine (16:0)[ce] + H2O[ce] => sn-glycero-3-phosphoethanolamine[ce] + H+[ce] + palmitate[ce] |  | PAPLA_05497 |
| r_3056 | LPE phospholipase B (18:0), cell envelope | 1-acylglycerophosphoethanolamine (18:0)[ce] + H2O[ce] => sn-glycero-3-phosphoethanolamine[ce] + H+[ce] + stearate[ce] |  | PAPLA_05497 |
| r_3062 | PS phospholipase B (1-16:0, 2-18:1), cell envelope | H2O[ce] + phosphatidyl-L-serine (1-16:0, 2-18:1)[ce] => 1-acylglycerophosphoserine (16:0)[ce] + H+[ce] + oleate[ce] |  | PAPLA_05497 |
| r_3064 | PS phospholipase B (1-18:0, 2-18:1), cell envelope | H2O[ce] + phosphatidyl-L-serine (1-18:0, 2-18:1)[ce] => 1-acylglycerophosphoserine (18:0)[ce] + H+[ce] + oleate[ce] |  | PAPLA_05497 |
| r_3066 | LPS phospholipase B (16:0), cell envelope | 1-acylglycerophosphoserine (16:0)[ce] + H2O[ce] => sn-glycero-3-phosphoserine[ce] + H+[ce] + palmitate[ce] |  | PAPLA_05497 |
| r_3068 | LPS phospholipase B (18:0), cell envelope | 1-acylglycerophosphoserine (18:0)[ce] + H2O[ce] => sn-glycero-3-phosphoserine[ce] + H+[ce] + stearate[ce] |  | PAPLA_05497 |
| r_3074 | PI phospholipase B (1-16:0, 2-18:1), cell envelope | 1-phosphatidyl-1D-myo-inositol (1-16:0, 2-18:1)[ce] + H2O[ce] => 1-acylglycerophosphoinositol (16:0)[ce] + H+[ce] + oleate[ce] |  | PAPLA_05497 |
| r_3076 | PI phospholipase B (1-18:0, 2-18:1), cell envelope | 1-phosphatidyl-1D-myo-inositol (1-18:0, 2-18:1)[ce] + H2O[ce] => 1-acylglycerophosphoinositol (18:0)[ce] + H+[ce] + oleate[ce] |  | PAPLA_05497 |
| r_3078 | LPI phospholipase B (16:0), cell envelope | 1-acylglycerophosphoinositol (16:0)[ce] + H2O[ce] => sn-glycero-3-phosphoinositol[ce] + 2 H+[ce] + palmitate[ce] |  | PAPLA_05497 |
| r_3080 | LPI phospholipase B (18:0), cell envelope | 1-acylglycerophosphoinositol (18:0)[ce] + H2O[ce] => sn-glycero-3-phosphoinositol[ce] + 2 H+[ce] + stearate[ce] |  | PAPLA_05497 |
| r_3094 | PI 4,5-P2 phospholipase C (1-16:0, 2-18:1), nucleus | H2O[n] + 1-phosphatidyl-1D-myo-inositol 4,5-bisphosphate (1-16:0, 2-18:1)[n] => 1D-myo-inositol 1,4,5-trisphosphate[n] + 6 H+[n] + diglyceride (1-16:0, 2-18:1)[n] |  | PAPLA_03893 |
| r_3096 | PI 4,5-P2 phospholipase C (1-18:0, 2-18:1), nucleus | H2O[n] + 1-phosphatidyl-1D-myo-inositol 4,5-bisphosphate (1-18:0, 2-18:1)[n] => 1D-myo-inositol 1,4,5-trisphosphate[n] + 6 H+[n] + diglyceride (1-18:0, 2-18:1)[n] |  | PAPLA_03893 |
| r_3102 | PG phospholipase C (1-16:0, 2-18:1), mitochondrial membrane | H2O[mm] + phosphatidylglycerol (1-16:0, 2-18:1)[mm] => 2 H+[mm] + glycerol 3-phosphate[mm] + diglyceride (1-16:0, 2-18:1)[mm] |  | PAPLA_05717 or PAPLA_06220 |
| r_3108 | PC phospholipase D (1-16:0, 2-18:1), cell envelope | H2O[ce] + phosphatidylcholine (1-16:0, 2-18:1)[ce] + 2 H+[ce] => choline[ce] + H+[ce] + phosphatidate (1-16:0, 2-18:1)[ce] |  | PAPLA_05089 or PAPLA_05090 or PAPLA_05214 |
| r_3110 | PC phospholipase D (1-18:0, 2-18:1), cell envelope | H2O[ce] + phosphatidylcholine (1-18:0, 2-18:1)[ce] + 2 H+[ce] => choline[ce] + H+[ce] + phosphatidate (1-18:0, 2-18:1)[ce] |  | PAPLA_05089 or PAPLA_05090 or PAPLA_05214 |
| r_3116 | PI 3-P phosphatase (1-16:0, 2-18:1), cell envelope | H2O[ce] + 1-phosphatidyl-1D-myo-inositol 3-phosphate (1-16:0, 2-18:1)[ce] => 1-phosphatidyl-1D-myo-inositol (1-16:0, 2-18:1)[ce] + phosphate[ce] + 2 H+[ce] |  | PAPLA_00633 |
| r_3118 | PI 3-P phosphatase (1-18:0, 2-18:1), cell envelope | H2O[ce] + 1-phosphatidyl-1D-myo-inositol 3-phosphate (1-18:0, 2-18:1)[ce] => 1-phosphatidyl-1D-myo-inositol (1-18:0, 2-18:1)[ce] + phosphate[ce] + 2 H+[ce] |  | PAPLA_00633 |
| r_3140 | PI 3-P phosphatase (1-16:0, 2-18:1), Golgi membrane | H2O[gm] + 1-phosphatidyl-1D-myo-inositol 3-phosphate (1-16:0, 2-18:1)[gm] => phosphate[gm] + 1-phosphatidyl-1D-myo-inositol (1-16:0, 2-18:1)[gm] + 2 H+[gm] |  | PAPLA_05592 |
| r_3142 | PI 3-P phosphatase (1-18:0, 2-18:1), Golgi membrane | H2O[gm] + 1-phosphatidyl-1D-myo-inositol 3-phosphate (1-18:0, 2-18:1)[gm] => phosphate[gm] + 1-phosphatidyl-1D-myo-inositol (1-18:0, 2-18:1)[gm] + 2 H+[gm] |  | PAPLA_05592 |
| r_3148 | PI 4-P phosphatase (1-16:0, 2-18:1), cell envelope | 1-phosphatidyl-1D-myo-inositol 4-phosphate (1-16:0, 2-18:1)[ce] + H2O[ce] => 1-phosphatidyl-1D-myo-inositol (1-16:0, 2-18:1)[ce] + phosphate[ce] + 2 H+[ce] |  | PAPLA_00633 |
| r_3150 | PI 4-P phosphatase (1-18:0, 2-18:1), cell envelope | 1-phosphatidyl-1D-myo-inositol 4-phosphate (1-18:0, 2-18:1)[ce] + H2O[ce] => 1-phosphatidyl-1D-myo-inositol (1-18:0, 2-18:1)[ce] + phosphate[ce] + 2 H+[ce] |  | PAPLA_00633 |
| r_3172 | PI 4-P phosphatase (1-16:0, 2-18:1), Golgi membrane | H2O[gm] + 1-phosphatidyl-1D-myo-inositol 4-phosphate (1-16:0, 2-18:1)[gm] => phosphate[gm] + 1-phosphatidyl-1D-myo-inositol (1-16:0, 2-18:1)[gm] + 2 H+[gm] |  | PAPLA_00633 or PAPLA_05592 |
| r_3174 | PI 4-P phosphatase (1-18:0, 2-18:1), Golgi membrane | H2O[gm] + 1-phosphatidyl-1D-myo-inositol 4-phosphate (1-18:0, 2-18:1)[gm] => phosphate[gm] + 1-phosphatidyl-1D-myo-inositol (1-18:0, 2-18:1)[gm] + 2 H+[gm] |  | PAPLA_00633 or PAPLA_05592 |
| r_3180 | PI 3,5-P2 phosphatase (1-16:0, 2-18:1), cell envelope | H2O[ce] + 1-phosphatidyl-1D-myo-inositol 3,5-bisphosphate (1-16:0, 2-18:1)[ce] => phosphate[ce] + 1-phosphatidyl-1D-myo-inositol 3-phosphate (1-16:0, 2-18:1)[ce] + 2 H+[ce] |  | PAPLA_00633 |
| r_3182 | PI 3,5-P2 phosphatase (1-18:0, 2-18:1), cell envelope | H2O[ce] + 1-phosphatidyl-1D-myo-inositol 3,5-bisphosphate (1-18:0, 2-18:1)[ce] => phosphate[ce] + 1-phosphatidyl-1D-myo-inositol 3-phosphate (1-18:0, 2-18:1)[ce] + 2 H+[ce] |  | PAPLA_00633 |
| r_3196 | PI 4,5-P2 phosphatase (1-16:0, 2-18:1), cell envelope | 1-phosphatidyl-1D-myo-inositol 4,5-bisphosphate (1-16:0, 2-18:1)[ce] + H2O[ce] => 1-phosphatidyl-1D-myo-inositol 4-phosphate (1-16:0, 2-18:1)[ce] + phosphate[ce] + 2 H+[ce] |  | PAPLA_00633 |
| r_3198 | PI 4,5-P2 phosphatase (1-18:0, 2-18:1), cell envelope | 1-phosphatidyl-1D-myo-inositol 4,5-bisphosphate (1-18:0, 2-18:1)[ce] + H2O[ce] => 1-phosphatidyl-1D-myo-inositol 4-phosphate (1-18:0, 2-18:1)[ce] + phosphate[ce] + 2 H+[ce] |  | PAPLA_00633 |
| r_3220 | PI 3,5-P2 phosphatase (1-16:0, 2-18:1), vacuolar membrane | H2O[vm] + 1-phosphatidyl-1D-myo-inositol 3,5-bisphosphate (1-16:0, 2-18:1)[vm] => phosphate[vm] + 1-phosphatidyl-1D-myo-inositol 3-phosphate (1-16:0, 2-18:1)[vm] + 2 H+[vm] |  | (PAPLA_03369 and PAPLA_05592) or (PAPLA_03369 and PAPLA_00633) |
| r_3222 | PI 3,5-P2 phosphatase (1-18:0, 2-18:1), vacuolar membrane | H2O[vm] + 1-phosphatidyl-1D-myo-inositol 3,5-bisphosphate (1-18:0, 2-18:1)[vm] => phosphate[vm] + 1-phosphatidyl-1D-myo-inositol 3-phosphate (1-18:0, 2-18:1)[vm] + 2 H+[vm] |  | (PAPLA_03369 and PAPLA_05592) or (PAPLA_03369 and PAPLA_00633) |
| r_3228 | DGPP phosphatase (1-16:0, 2-18:1), vacuolar membrane | H2O[vm] + 1,2-diacylglycerol 3-diphosphate (1-16:0, 2-18:1)[vm] => phosphate[vm] + phosphatidate (1-16:0, 2-18:1)[vm] + 2 H+[vm] |  | PAPLA_05340 |
| r_3230 | DGPP phosphatase (1-18:0, 2-18:1), vacuolar membrane | H2O[vm] + 1,2-diacylglycerol 3-diphosphate (1-18:0, 2-18:1)[vm] => phosphate[vm] + phosphatidate (1-18:0, 2-18:1)[vm] + 2 H+[vm] |  | PAPLA_05340 |
| r_3244 | lysoPA phosphatase (16:0), vacuolar membrane | H2O[vm] + 1-acyl-sn-glycerol 3-phosphate (16:0)[vm] => phosphate[vm] + 1-monoglyceride (16:0)[vm] + 2 H+[vm] |  | PAPLA_05340 |
| r_3246 | lysoPA phosphatase (18:0), vacuolar membrane | H2O[vm] + 1-acyl-sn-glycerol 3-phosphate (18:0)[vm] => phosphate[vm] + 1-monoglyceride (18:0)[vm] + 2 H+[vm] |  | PAPLA_05340 |
| r_3344 | PA kinase (1-16:0, 2-18:1), vacuolar membrane | phosphatidate (1-16:0, 2-18:1)[vm] + ATP[vm] + H+[vm] <=> ADP[vm] + 1,2-diacylglycerol 3-diphosphate (1-16:0, 2-18:1)[vm] |  |  |
| r_3346 | PA kinase (1-18:0, 2-18:1), vacuolar membrane | phosphatidate (1-18:0, 2-18:1)[vm] + ATP[vm] + H+[vm] <=> ADP[vm] + 1,2-diacylglycerol 3-diphosphate (1-18:0, 2-18:1)[vm] |  |  |
| r_4041 | biomass pseudoreaction | 154.1 ATP[c] + 154.1 H2O[c] + lipid[c] + 0.00099 riboflavin[c] + 0.02 sulphate[c] + 1e-06 heme a[c] + protein[c] + carbohydrate[c] + RNA[c] + DNA[c] => 154.1 ADP[c] + biomass[c] + 154.1 H+[c] + 154.1 phosphate[c] |  |  |
| r_4042 | raffinose invertase | raffinose[e] + H2O => D-fructose[e] + melibiose[e] | 5.3.1.16 | PAPLA_03678 |
| r_4047 | protein pseudoreaction | 0.14099 Ala-tRNA(Ala)[c] + 0.13545 Arg-tRNA(Arg)[c] + 0.11251 Asn-tRNA(Asn)[c] + 0.13154 Asp-tRNA(Asp)[c] + 0.051318 Cys-tRNA(Cys)[c] + 0.12113 Gln-tRNA(Gln)[c] + 0.13088 Glu-tRNA(Glu)[c] + 0.13802 Gly-tRNA(Gly)[c] + 0.097893 His-tRNA(His)[c] + 0.12567 Ile-tRNA(Ile)[c] + 0.13922 Leu-tRNA(Leu)[c] + 0.12316 Lys-tRNA(Lys)[c] + 0.096124 Met-tRNA(Met)[c] + 0.11341 Phe-tRNA(Phe)[c] + 0.13248 Pro-tRNA(Pro)[c] + 0.13862 Ser-tRNA(Ser)[c] + 0.13412 Thr-tRNA(Thr)[c] + 0.071888 Trp-tRNA(Trp)[c] + 0.098995 Tyr-tRNA(Tyr)[c] + 0.13594 Val-tRNA(Val)[c] => 0.14099 tRNA(Ala)[c] + 0.13545 tRNA(Arg)[c] + 0.11251 tRNA(Asn)[c] + 0.13154 tRNA(Asp)[c] + 0.051318 tRNA(Cys)[c] + 0.12113 tRNA(Gln)[c] + 0.13088 tRNA(Glu)[c] + 0.13802 tRNA(Gly)[c] + 0.097893 tRNA(His)[c] + 0.12567 tRNA(Ile)[c] + 0.13922 tRNA(Leu)[c] + 0.12316 tRNA(Lys)[c] + 0.096124 tRNA(Met)[c] + 0.11341 tRNA(Phe)[c] + 0.13248 tRNA(Pro)[c] + 0.13862 tRNA(Ser)[c] + 0.13412 tRNA(Thr)[c] + 0.071888 tRNA(Trp)[c] + 0.098995 tRNA(Tyr)[c] + 0.13594 tRNA(Val)[c] + protein[c] |  |  |
| r_4048 | carbohydrate pseudoreaction | 0.14394 (1->3)-beta-D-glucan[ce] + 0.14394 (1->6)-beta-D-glucan[ce] + 0.63086 glycogen[c] + 0.029893 mannan[c] + 0.2016 trehalose[c] => carbohydrate[c] |  |  |
| r_4049 | RNA pseudoreaction | 0.0038342 AMP[c] + 0.0038199 CMP[c] + 0.0038323 GMP[c] + 0.00383 UMP[c] => RNA[c] |  |  |
| r_4050 | DNA pseudoreaction | 0.0012246 dAMP[c] + 0.0011363 dCMP[c] + 0.0012843 dGMP[c] + 0.0011913 dTMP[c] => DNA[c] |  |  |
| r_4063 | lipid backbone pseudoreaction | 0.00094028 1-phosphatidyl-1D-myo-inositol backbone[c] + 0.011743 ergosterol[c] + 0.021532 fatty acid backbone[c] + 0.0014687 phosphatidylcholine backbone[c] + 0.0011346 phosphatidylethanolamine backbone[c] + 0.13144 triglyceride backbone[c] + 0.0037223 phosphatidyl-L-serine backbone[erm] + 0.0088662 diglyceride backbone[c] + 0.00017096 cardiolipin backbone[mm] => lipid backbone[c] |  |  |
| r_4065 | lipid chain pseudoreaction | 0.060834 C18:2 chain[c] + 0.041602 C16:0 chain[c] + 0.0091616 C18:0 chain[c] + 0.048847 C18:1 chain[c] => lipid chain[c] |  |  |
| r_4170 | UDP-N-acetyl-D-glucosamine:dolichyl-phosphate N-acetyl-D-glucosamine phosphotransferase | dolichyl phosphate[er] + UDP-N-acetyl-alpha-D-glucosamine[er] <=> N-Acetyl-D-glucosaminyldiphosphodolichol[er] + UMP[er] | 2.7.8.15 | PAPLA_02162 |
| r_4184 | 4-hydroxy-4-methyl-2-oxoglutarate pyruvate-lyase (pyruvate-forming) | 4-hydroxy-4-methyl-2-oxoglutarate[c] <=> 2 pyruvate[c] | 4.1.3.17;4.1.1.3 | PAPLA_03009 |
| r_4186 | L-methionine:thioredoxin-disulfide S-oxidoreductase | H2O[c] + L-methionine[c] + TRX1 disulphide[c] <=> TRX1[c] + L-methionine (S)-S-oxide[c] | 1.8.4.11 | PAPLA_06381 |
| r_4202 | L-methionine:oxidized-thioredoxin S-oxidoreductase | H2O[c] + L-methionine[c] + TRX1 disulphide[c] <=> TRX1[c] + L-Methionine S-oxide[c] | 1.8.4.14 | PAPLA_03212 |
| r_4218 | D-aminoacyl-tRNA deacylase (DTD) (EC 3.1.1.96) (D-tyrosyl-tRNA(Tyr) deacylase) (Gly-tRNA(Ala) deacylase) (EC 3.1.1.-) | H2O[c] + Glycyl-tRNA(Ala)[c] <=> L-glycine[c] + tRNA(Ala)[c] | 3.1.1.-;3.1.1.96 | PAPLA_03939 |
| r_4219 | D-aminoacyl-tRNA deacylase (DTD) (EC 3.1.1.96) (D-tyrosyl-tRNA(Tyr) deacylase) (Gly-tRNA(Ala) deacylase) (EC 3.1.1.-) | H2O[c] + D-tyrosyl-tRNA(Tyr)[c] <=> H+[c] + tRNA(Tyr)[c] + D-tyrosine[c] | 3.1.1.-;3.1.1.96 | PAPLA_03939 |
| r_4245 | 2-O-(6-phospho-alpha-D-mannosyl)-D-glycerate 6-phosphomannohydrolase | H2O[v] + 2-O-(6-phospho-alpha-D-mannosyl)-D-glycerate[v] + 2 H+[v] => D-mannose 6-phosphate[v] + D-Glycerate[v] | 3.2.1.24 | PAPLA_05316 or PAPLA_05317 or PAPLA_06091 or PAPLA_06365 |
| r_4252 | Thiamine thiazole synthase (Thiazole biosynthetic enzyme) | L-glycine[c] + NAD[c] + Sulfur donor[c] <=> 3 H2O[c] + nicotinamide[c] + ADP-5-ethyl-4-methylthiazole-2-carboxylate[c] |  |  |
| r_4260 | Deaminated glutathione amidase (dGSH amidase) (EC 3.5.1.-) (Nitrilase homolog 1) | H2O[c] + N-(4-oxoglutarate)-L-cysteinylglycine[c] <=> 2-oxoglutarate[c] + L-cysteinylglycine[c] | 3.5.1.- | PAPLA_00778 |
| r_4261 | Deaminated glutathione amidase (dGSH amidase) (EC 3.5.1.-) (Nitrilase homolog 1) | H2O[m] + N-(4-oxoglutarate)-L-cysteinylglycine[m] <=> 2-oxoglutarate[m] + L-cysteinylglycine[m] | 3.5.1.- | PAPLA_00778 |
| r_4267 | 2'-Deoxyguanosine 5'-triphosphate diphosphohydrolase | dGTP[c] + H2O[c] => dGMP[c] + diphosphate[c] + 2 H+[c] | 3.6.1.19;3.6.1.9 | PAPLA_04480 |
| r_4268 | dTTP diphosphohydrolase | dTTP[c] + H2O[c] + H+[c] => diphosphate[c] + dTMP[c] + H+[c] | 3.6.1.9 | PAPLA_04480 |
| r_4275 | Fe(II):NADP+ oxidoreductase | NADPH[c] + 2 iron(3+)[e] <=> H+[c] + 2 iron(2+)[c] + NADP(+)[c] | 1.16.1.7 | PAPLA_01625 or PAPLA_02817 |
| r_4276 | Fe(II):NADP+ oxidoreductase | NADPH[c] + 2 iron(3+)[v] <=> H+[c] + 2 iron(2+)[c] + NADP(+)[c] | 1.16.1.7 | PAPLA_01625 or PAPLA_02817 |
| r_4280 | octanoyl-[acp]:protein N6-octanoyltransferase | octanoyl-ACP[m] + Apoprotein[m] <=> ACP1[m] + Protein N6-(octanoyl)lysine[m] | 2.3.1.181 | PAPLA_00440 |
| r_4281 | lipoyl-[acp]:protein N6-lipoyltransferase | Apoprotein[m] + Lipoyl-[acp][m] <=> ACP1[m] + Protein N6-(lipoyl)lysine[m] | 2.3.1.181 | PAPLA_00440 |
| y200001 | glycinamide ribonucleotide transformylase | 5-phospho-ribosyl-glycineamide[c] + ATP[c] + formate[c] => 5'-phosphoribosyl-N-formylglycineamide[c] + ADP[c] + phosphate[c] + H+[c] | 2.1.2.- | PAPLA_04906 |
| y200002 | mannitol dehydrogenase | D-mannitol[c] + NADP(+)[c] <=> D-fructose[c] + H+[c] + NADPH[c] | 1.1.1.138 | PAPLA_01438 or PAPLA_04129 |
| y200008 | O-succinylhomoserine lyase (elimination) | O-succinyl-L-homoserine[c] + H+[c] => 2-oxobutanoate[c] + ammonium[c] + succinate[c] | 2.5.1.48 | PAPLA_00550 |
| y200015 | ATP:pantothenate 4'-phosphotransferase | pantetheine[c] + ATP[c] => ADP[c] + H+[c] + pantetheine 4'-phosphate[c] | 2.7.1.33 | PAPLA_02884 |
| y200016 | ATP:pantothenate 4'-phosphotransferase (reação incompleta e diferente) | (R)-pantothenate[c] + ATP[c] + 3 H+[c] => (R)-4'-phosphopantothenic acid[c] + ADP[c] + H+[c] | 2.7.1.33 | PAPLA_02884 |
| y200017 | UDP-glucose:NAD+ 6-oxidoreductase | H2O[c] + 2 NAD[c] + UDP-D-glucose[c] <=> UDP-D-glucuronate[c] + 3 H+[c] + 2 NADH[c] | 1.1.1.22 | PAPLA_04775 |
| y200019 | ADP-ribose ribophosphohydrolase | H2O[c] + ADP-ribose[c] + 2 H+[c] => AMP[c] + 2 H+[c] + ribose-5-phosphate[c] | 3.6.1.13 | PAPLA_00737 |
| y200020 | ribonucleotide reductase | UDP[c] + TRX1[c] => TRX1-disulphide[c] + dUDP[c] + H2O[c] | 1.17.4.1 | PAPLA_00211 |
| y200021 | ribonucleotide reductase | UDP[n] + TRX1[n] => TRX1-disulphide[n] + dUDP[n] + H2O[c] | 1.17.4.1 | PAPLA_00211 |
| y200029 | dGTP:uridine 5'-phosphotransferase | dGTP[c] + uridine[c] <=> dGDP[c] + 2 H+[c] + UMP[c] | 2.7.1.48 | PAPLA_00418 or PAPLA_04844 |
| y200030 | dGTP:cytidine 5'-phosphotransferase | cytidine[c] + dGTP[c] <=> CMP[c] + dGDP[c] + 2 H+[c] | 2.7.1.48 | PAPLA_00418 or PAPLA_04844 |
| y200031 | dTTP:cytidine 5'-phosphotransferase | cytidine[c] + dTTP[c] <=> CMP[c] + dTDP[c] + 2 H+[c] | 2.7.1.48 | PAPLA_00418 or PAPLA_04844 |
| y200032 | dTTP:uridine 5'-phosphotransferase | dTTP[c] + uridine[c] <=> dTDP[c] + 2 H+[c] + UMP[c] | 2.7.1.48 | PAPLA_00418 or PAPLA_04844 |
| y200033 | dCTP:uridine 5'-phosphotransferase | dCTP[c] + uridine[c] <=> dCDP[c] + 2 H+[c] + UMP[c] | 2.7.1.48 | PAPLA_00418 or PAPLA_04844 |
| y200034 | dUTP:uridine 5'-phosphotransferase | dUTP[c] + uridine[c] <=> dUDP[c] + 2 H+[c] + UMP[c] | 2.7.1.48 | PAPLA_00418 or PAPLA_04844 |
| y200035 | dCTP:cytidine 5'-phosphotransferase | cytidine[c] + dCTP[c] <=> CMP[c] + dCDP[c] + 2 H+[c] | 2.7.1.48 | PAPLA_00418 or PAPLA_04844 |
| y200036 | dUTP:cytidine 5'-phosphotransferase | cytidine[c] + dUTP[c] <=> CMP[c] + dUDP[c] + 2 H+[c] | 2.7.1.48 | PAPLA_00418 or PAPLA_04844 |
| y300004 | long-chain alcohol dehydrogenase (C10) | decanol[c] + NAD[c] <=> decanal[c] + H+[c] + 2 NADH[c] |  | PAPLA_02635 or PAPLA_04110 or PAPLA_04347 |
| y300006 | long-chain alcohol dehydrogenase (C12) | dodecanol[c] + NAD[c] <=> dodecanal[c] + H+[c] + 2 NADH[c] |  | PAPLA_02635 or PAPLA_04110 or PAPLA_04347 |
| y300008 | long-chain alcohol dehydrogenase (C16) | hexadecanol[c] + H2O[c] + 2 NAD[c] <=> hexadecanal[c] + 2 H+[c] + 2 NADH[c] |  | PAPLA_02635 or PAPLA_04110 or PAPLA_04347 |
| y300009 | oleoyl-CoA desaturase (n-C18:1CoA - n-C18:2CoA), ER membrane | 5 H+[erm] + oxygen[erm] + NADH[erm] + oleoyl-CoA[erm] => linoleoyl-CoA[erm] + 2 H2O[erm] + NAD[erm] | 1.14.19.1 |  |
| y300025 | long-chain aldehyde dehydrogenase (C10) | decanal[c] + H2O[c] + NAD[c] => decanoate[c] + 2 H+[c] + NADH[c] | 1.2.1.48 | PAPLA_02635 or PAPLA_04110 or PAPLA_04347 or PAPLA_05275 |
| y300026 | long-chain aldehyde dehydrogenase (C12) | dodecanal[c] + H2O[c] + NAD[c] => decanoate[c] + 2 H+[c] + NADH[c] | 1.2.1.48 | PAPLA_02635 or PAPLA_04110 or PAPLA_04347 |
| y300031 | xanthine dehydrogenase | 9H-xanthine[c] + H2O[c] + NAD[c] => urate[c] + H+[c] + NADH[c] | 1.17.1.4 | PAPLA_05190 |
| y300032 | urate oxidase | urate[c] + H2O[c] + oxygen[c] => 5-hydroxyisourate[c] + hydrogen peroxide[c] | 1.7.3.3 | PAPLA_06113 |
| y300040 | erythrose reductase | erythrose[c] + H+[c] + NADH[c] <=> erythritol[c] + NAD[c] | 1.1.1.21 | PAPLA_01202 or PAPLA_02343 |
| y300044 | 4-methyl-2-oxopentanoate dehydrogenase | 4-methyl-2-oxopentanoate[m] + coenzyme A[m] + NAD[m] + 4 H+[m] => 3-methylbutanoyl-CoA[m] + carbon dioxide[m] + NADH[m] | 1.2.1.- | (PAPLA_05058 and PAPLA_02484 and PAPLA_04542) or (PAPLA_05058 and PAPLA_02484 and PAPLA_04816) |
| y300045 | 3-methyl-2-oxopentanoate dehydrogenase | 3-methyl-2-oxobutanoate[m] + coenzyme A[m] + NAD[m] + 4 H+[m] => 2-methylpropanoyl-CoA[m] + carbon dioxide[m] + NADH[m] | 1.2.1.- | (PAPLA_05058 and PAPLA_02484 and PAPLA_04542) or (PAPLA_05058 and PAPLA_02484 and PAPLA_04816) |
| y300046 | (S)3-methyl-2-oxopentanoate dehydrogenase | (S)-3-methyl-2-oxopentanoate[m] + coenzyme A[m] + NAD[m] + 4 H+[m] => (S)-2-methylbutanoyl-CoA[m] + carbon dioxide[m] + NADH[m] | 1.2.1.- | (PAPLA_05058 and PAPLA_02484 and PAPLA_04542) or (PAPLA_05058 and PAPLA_02484 and PAPLA_04816) |
| y300050 | hydroxymethylglutaryl-CoA lyase | 3-hydroxy-3-methylglutaryl-CoA[m] => 5H+[m] + acetoacetate[m] + acetyl-CoA[m] | 4.1.3.4 | PAPLA_00944 |
| y300051 | 2-oxoadipate dehydrogenase | 2-oxoadipic acid[m] + coenzyme A[m] + NAD[m] + 5 H+[m] => glutaryl-CoA[m] + carbon dioxide[m] + NADH[m] | 1.3.8.6 | PAPLA_04942 |
| y300052 | glutaryl-CoA dehydrogenase | glutaryl-CoA[m] + FAD[m] + H+[m] => crotonoyl-CoA[m] + carbon dioxide[m] + FADH2[m] |  | (PAPLA_03721 and PAPLA_03685 and PAPLA_02484) or (PAPLA_01932 and PAPLA_03685 and PAPLA_02484) |
| y300055 | (S)-3-Hydroxybutanoyl-CoA oxidoreductase | (S)-3-hydroxybutanoyl-CoA[m] + NAD[m] => acetoacetyl-CoA[m] + 9 H+[m] + NADH[m] | 1.1.1.35 | PAPLA_06102 or PAPLA_06333 |
| y300056 | cellobiose glucohydrolase | cellobiose[c] + H2O[c] => 2 D-glucose[c] | 3.2.1.21 | PAPLA_00291 or PAPLA_00872 or PAPLA_02750 or PAPLA_04198 |
| y300058 | 5-oxo-L-proline amidohydrolase (ATP-hydrolysing) | 5-oxoproline[c] + ATP[c] + 2 H2O[c] => H+[c] + ADP[c] + L-glutamate[c] + phosphate[c] | 3.5.2.9 | PAPLA_05383 or PAPLA_06045 or PAPLA_06046 |
| y300059 | succinyl-CoA:acetoacetate CoA-transferase | acetoacetate[m] + succinyl-CoA[m] <=> acetoacetyl-CoA[m] + succinate[m] | 2.8.3.5 | PAPLA_02254 |
| y300060 | GTP diphosphohydrolase (diphosphate-forming); | GTP[c] + H2O[c] => diphosphate[c] + GMP[c] + 2 H+[c] | 3.6.1.19 | PAPLA_04480 |
| y300061 | GTP diphosphate-lyase (cyclizing; 3',5'-cyclic-GMP-forming) | GTP[c] + H2O[c] => 3',5'-cyclic GMP[c] + diphosphate[c] + 3 H+[c] | 4.6.1.1 | PAPLA_06334 |
| y300065 | L-Serine:pyruvate aminotransferase | L-serine[c] + pyruvate[c] <=> hydroxypyruvate[c] + L-alanine[c] | 2.6.1.51 | PAPLA_04226 |
| y300066 | L-Serine:glyoxylate aminotransferase | glyoxylate[c] + L-serine[c] <=> hydroxypyruvate[c] + L-glycine[c] | 2.6.1.45 | PAPLA_04226 |
| y300067 | serine racemase | L-serine[c] <=> D-serine[c] | 5.1.1.18 | PAPLA_02126 |
| y300068 | L-Serine hydro-lyase | L-serine[c] => dehydroalanine[c] + H2O[c] | 4.3.1.17 | PAPLA_00968 |
| y300072 | ATP:D-glucosamine 6-phosphotransferase | D-glucosamine[c] + ATP[c] + H+[c] => ADP[c] + alpha-D-glucosamine 6-phosphate[c] + H+[c] | 2.7.1.1 | PAPLA_01860 or PAPLA_03777 |
| y300073 | N-Acetyl-D-glucosamine-6-phosphate amidohydrolase | H2O[c] + N-acetyl-D-glucosamine 6-phosphate[c] + H+[c] => acetate[c] + alpha-D-glucosamine 6-phosphate[c] | 3.5.1.25 | PAPLA_03027 |
| y300074 | dTDP phosphohydrolase | dTDP[c] + H2O[c] + 2 H+[c] => dTMP[c] + H+[c] + phosphate[c] | 3.6.1.5 | PAPLA_05894 |
| y300075 | dTTP nucleotidohydrolase | dTTP[c] + H2O[c] => dTDP[c] + 2 H+[c] + phosphate[c] | 3.6.1.5 | PAPLA_05894 |
| y300078 | 2'-Deoxyinosine-5'-triphosphate pyrophosphohydrolase | dITP[c] + H2O[c] => 2'-deoxyinosine-5-phosphate[c] + diphosphate[c] + H+[c] | 3.6.1.19 | PAPLA_04480 |

# Supplementary Table S2 - Design of Experiments (CCRD) for *Papiliotrema laurentii* cultivation. Fermentation runs (n = 20) showing the variable levels tested (microbial inoculum, C:N ratio, and kLa, represented by MV:FV) and the experimental responses (biomass concentration, lipid titer, and lipid content).

| Run | Inoculum (OD600) | C:N ratio (g/g) | (MV:FV) | Biomass (g/L) | Lipid titer (g/L) | Lipid content (%) |
| --- | --- | --- | --- | --- | --- | --- |
| 1 | 0.242 | 68.11 | 0.141 | 8.06 | 2.22 | 27.57 |
| 2 | 0.658 | 68.11 | 0.141 | 8.62 | 2.37 | 27.50 |
| 3 | 0.242 | 91.89 | 0.141 | 8.35 | 2.86 | 34.22 |
| 4 | 0.658 | 91.89 | 0.141 | 8.06 | 2.43 | 30.09 |
| 5 | 0.242 | 68.11 | 0.259 | 6.39 | 1.75 | 27.46 |
| 6 | 0.658 | 68.11 | 0.259 | 6.82 | 1.77 | 25.99 |
| 7 | 0.242 | 91.89 | 0.259 | 6.05 | 1.69 | 28.01 |
| 8 | 0.658 | 91.89 | 0.259 | 6.46 | 1.94 | 30.05 |
| 9 | 0.100 | 80.00 | 0.200 | 6.87 | 2.14 | 31.13 |
| 10 | 0.800 | 80.00 | 0.200 | 7.44 | 2.16 | 28.98 |
| 11 | 0.450 | 60.00 | 0.200 | 7.28 | 2.01 | 27.59 |
| 12 | 0.450 | 100.00 | 0.200 | 6.52 | 2.05 | 31.42 |
| 13 | 0.450 | 80.00 | 0.100 | 9.58 | 2.75 | 28.74 |
| 14 | 0.450 | 80.00 | 0.300 | 5.90 | 1.57 | 26.55 |
| 15 | 0.450 | 80.00 | 0.200 | 7.53 | 1.99 | 26.48 |
| 16 | 0.450 | 80.00 | 0.200 | 7.03 | 1.93 | 27.51 |
| 17 | 0.450 | 80.00 | 0.200 | 7.08 | 2.10 | 29.68 |
| 18 | 0.450 | 80.00 | 0.200 | 7.22 | 2.06 | 27.89 |
| 19 | 0.450 | 80.00 | 0.200 | 7.22 | 2.01 | 27.91 |
| 20 | 0.450 | 80.00 | 0.200 | 7.19 | 2.01 | 27.92 |

# Supplementary Table S2a - ANOVA for biomass concentration (g/L) in CCRD. DF = degrees of freedom; Adj. SS = adjusted sum of squares; Adj. MS = adjusted mean square; F-value = Fisher’s statistic (variance ratio); Prob > F = probability (p-value) associated with the F-test. MV:FV = ratio of volume of the medium to volume of the flask. OD = initial microbial inoculum optical density at 600 nm.

| Source of variation | DF | Adj. SS | Adj. MS | F-value | Prob > F |
| --- | --- | --- | --- | --- | --- |
| Model (selected) | 9 | 15.1464 | 1.6829 | 42.11 | <0.001 |
| Linear | 3 | 14.1910 | 4.7303 | 118.36 | <0.001 |
| Inoculum (OD) | 1 | 0.3178 | 0.3178 | 7.95 | 0.018 |
| C:N ratio | 1 | 0.3731 | 0.3731 | 9.34 | 0.012 |
| (MV:FV) | 1 | 13.5001 | 13.5001 | 337.79 | <0.001 |
| Quadratic | 3 | 0.7931 | 0.2644 | 6.61 | 0.010 |
| Two-factor interaction | 3 | 0.1623 | 0.0541 | 1.35 | 0.312 |
| Error | 10 | 0.3997 | 0.0400 | — | — |
| Lack of fit | 5 | 0.2481 | 0.0496 | 1.64 | 0.301 |
| Pure error | 5 | 0.1516 | 0.0303 | — | — |
| Total | 19 | 15.5461 | — | — | — |

# Supplementary Table S2b - ANOVA for lipid titer (g/L) in CCRD. DF = degrees of freedom; Adj. SS = adjusted sum of squares; Adj. MS = adjusted mean square; F-value = Fisher’s statistic (variance ratio); Prob > F = probability (p-value) associated with the F-test. MV:FV = ratio of volume of the medium to volume of the flask. OD = initial microbial inoculum optical density at 600 nm.

| Source of variation | DF | Adj. SS | Adj. MS | F-value | Prob > F |
| --- | --- | --- | --- | --- | --- |
| Model (selected) | 9 | 1.8470 | 0.2052 | 16.30 | <0.001 |
| Linear | 3 | 1.6814 | 0.5605 | 44.51 | <0.001 |
| Inoculum (OD) | 1 | 0.0000 | 0.0000 | 0.00 | 0.975 |
| C:N ratio | 1 | 0.0545 | 0.0545 | 4.33 | 0.064 |
| (MV:FV) | 1 | 1.6269 | 1.6269 | 129.20 | <0.001 |
| Quadratic | 3 | 0.0691 | 0.0230 | 1.83 | 0.206 |
| Two-factor interaction | 3 | 0.0965 | 0.0322 | 2.56 | 0.114 |
| Error | 10 | 0.1259 | 0.0126 | — | — |
| Lack of fit | 5 | 0.1094 | 0.0219 | 6.60 | 0.029 |
| Pure error | 5 | 0.0166 | 0.0033 | — | — |
| Total | 19 | 1.9729 | — | — | — |

# Supplementary Table S2c - ANOVA for lipid content (%) in CCRD. DF = degrees of freedom; Adj. SS = adjusted sum of squares; Adj. MS = adjusted mean square; F-value = Fisher’s statistic (variance ratio); Prob > F = probability (p-value) associated with the F-test. MV:FV = ratio of volume of the medium to volume of the flask. OD = initial microbial inoculum optical density at 600 nm.

| Source of variation | DF | Adj. SS | Adj. MS | F-value | Prob > F |
| --- | --- | --- | --- | --- | --- |
| Model (selected) | 9 | 60.2318 | 6.6924 | 4.64 | 0.012 |
| Linear | 3 | 43.7215 | 14.5738 | 10.11 | 0.002 |
| Inoculum (OD) | 1 | 3.8517 | 3.8517 | 2.67 | 0.133 |
| C:N ratio | 1 | 30.1058 | 30.1058 | 20.89 | 0.001 |
| (MV:FV) | 1 | 9.7640 | 9.7640 | 6.77 | 0.026 |
| Quadratic | 3 | 10.9225 | 3.6408 | 2.53 | 0.117 |
| Two-factor interaction | 3 | 5.5878 | 1.8626 | 1.29 | 0.330 |
| Error | 10 | 14.4123 | 1.4412 | — | — |
| Lack of fit | 5 | 9.0504 | 1.8101 | 1.69 | 0.290 |
| Pure error | 5 | 5.3619 | 1.0724 | — | — |
| Total | 19 | 74.6441 | — | — | — |

# Supplementary Table S3 - Regression equations fitted to CCRD data in uncoded units.

| Response | Polynomial regression model |
| --- | --- |
| Biomass (g/L) | 7.12 + 3.17·Inoculum + 0.127·C:N – 36.2·kLa – 0.04·Inoculum² – 0.000655·(C:N)² + 58.0·kLa² – 0.044·Inoculum·C:N + 5.78·Inoculum·kLa – 0.079·C:N·kLa |
| Lipid titer (g/L) | 2.08 – 0.69·Inoculum + 0.027·C:N – 6.04·kLa + 1.13·Inoculum² + 0.000048·(C:N)² + 15.10·kLa² – 0.018·Inoculum·C:N + 5.55·Inoculum·kLa – 0.104·C:N·kLa |
| Lipid content (%) | 36.7 – 24.6·Inoculum – 0.273·C:N + 46.6·kLa + 16.3·Inoculum² + 0.0036·(C:N)² – 42.1·kLa² – 0.028·Inoculum·C:N + 48.2·Inoculum·kLa – 0.821·C:N·kLa |
