# Supplementary figures and images for: Improvement of the production of biomass and lipid by Papiliotrema laurentii combining flux balance analysis and central composite rotational design
Source: Bioprocess Biosyst Eng. 2026 Mar 23;49(5):1233–48. doi: 10.1007/s00449-026-03311-z (PMC13263264; doi:10.1007/s00449-026-03311-z)

## Plateau Model: RPM vs kLa

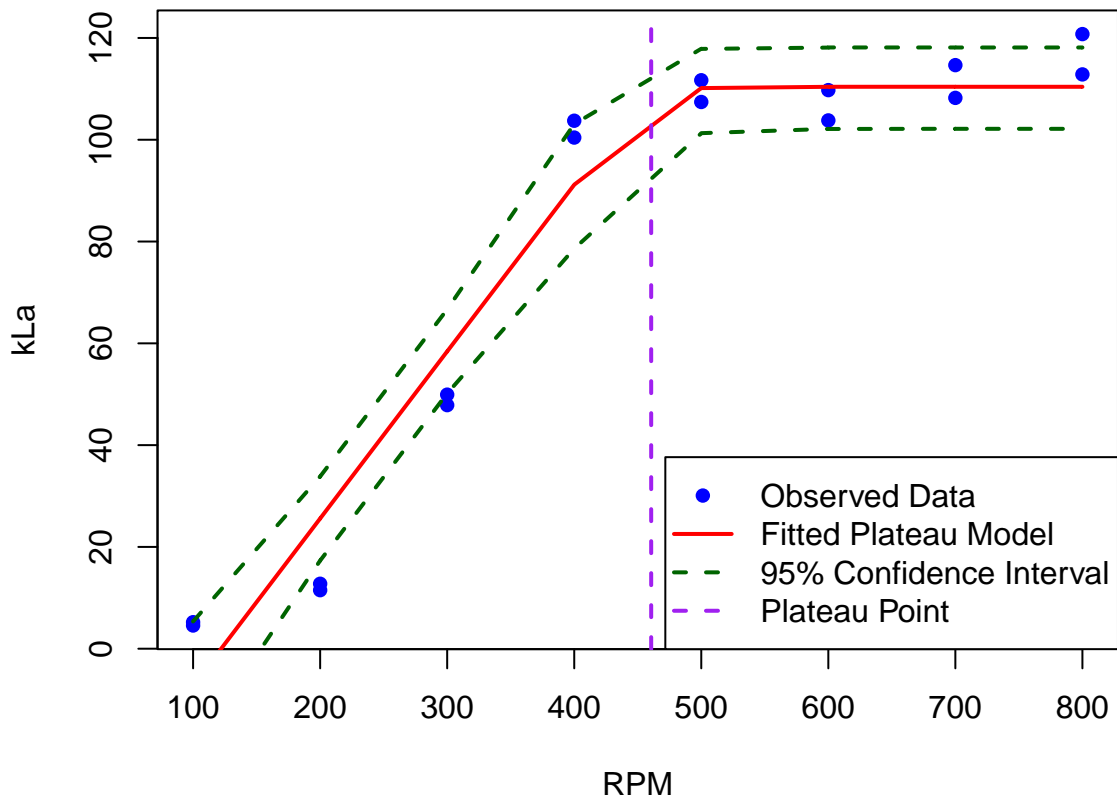

Supplement: Supplementary file 2 — Supplementary file2 (PDF 6 KB) [file 449_2026_3311_MOESM2_ESM.pdf]
